# An integrated approach of network pharmacology, molecular docking, and experimental verification uncovers kaempferol as the effective modulator of HSD17B1 for treatment of endometrial cancer

**DOI:** 10.1186/s12967-023-04048-z

**Published:** 2023-03-17

**Authors:** Guan-Yu Ruan, Li-Xiang Ye, Jian-Song Lin, Hong-Yu Lin, Li-Rui Yu, Cheng-Yan Wang, Xiao-Dan Mao, Shui-Hua Zhang, Peng-Ming Sun

**Affiliations:** 1 Laboratory of Gynecologic Oncology, Fujian Maternity and Child Health Hospital, College of Clinical Medicine for Obstetrics & Gynecology and Pediatrics, Fujian Medical University, Fuzhou, 350001 Fujian People’s Republic of China; 2 Fujian Key Laboratory of Women and Children’s Critical Diseases Research, Fujian Maternity and Child Health Hospital, Fuzhou, 350001 Fujian People’s Republic of China; 3 Fujian Clinical Research Center for Gynecologic Oncology, Fujian Maternity and Child Health Hospital (Fujian Obstetrics and Gynecology Hospital), Fuzhou, 350001 Fujian People’s Republic of China; 4 Fujian Center for Safety Evaluation of New Drugs, Fujian Medical University, No.1 Xue Fu Bei Road, University Town, Fuzhou, 350001 Fujian People’s Republic of China; 5 Department of Pathology, Fujian Maternity and Child Health Hospital, College of Clinical Medicine for Obstetrics & Gynecology and Pediatrics, Fujian Medical University, Fuzhou, 350001 Fujian People’s Republic of China; 6 Collage of Pharmacy, Fujian Medical University, Fuzhou, 351004 Fujian People’s Republic of China; 7 Animal Research Institute, Fujian Medical University, Fuzhou, 351004 Fujian People’s Republic of China

**Keywords:** Kaempferol, Human endometrial cancer, HSD17B1, Estrogen receptor α, Nude mice

## Abstract

**Background:**

Endometrial cancer (EC) is one of the most common gynecological malignancies globally, and the development of innovative, effective drugs against EC remains a key issue. Phytoestrogen kaempferol exhibits anti-cancer effects, but the action mechanisms are still unclear.

**Method:**

MTT assays, colony-forming assays, flow cytometry, scratch healing, and transwell assays were used to evaluate the proliferation, apoptosis, cell cycle, migration, and invasion of both ER-subtype EC cells. Xenograft experiments were used to assess the effects of kaempferol inhibition on tumor growth. Next-generation RNA sequencing was used to compare the gene expression levels in vehicle-treated versus kaempferol-treated Ishikawa and HEC-1-A cells. A network pharmacology and molecular docking technique were applied to identify the anti-cancer mechanism of kaempferol, including the building of target-pathway network. GO analysis and KEGG pathway enrichment analysis were used to identify cancer-related targets. Finally, the study validated the mRNA and protein expression using real-time quantitative PCR, western blotting, and immunohistochemical analysis.

**Results:**

Kaempferol was found to suppress the proliferation, promote apoptosis, and limit the tumor-forming, scratch healing, invasion, and migration capacities of EC cells. Kaempferol inhibited tumor growth and promotes apoptosis in a human endometrial cancer xenograft mouse model. No significant toxicity of kaempferol was found in human monocytes and normal cell lines at non-cytotoxic concentrations. No adverse effects or significant changes in body weight or organ coefficients were observed in 3–7 weeks’ kaempferol-treated animals. The RNA sequencing, network pharmacology, and molecular docking approaches identified the overall survival-related differentially expressed gene HSD17B1. Interestingly, kaempferol upregulated HSD17B1 expression and sensitivity in ER-negative EC cells. Kaempferol differentially regulated PPARG expression in EC cells of different ER subtypes, independent of its effect on ESR1. HSD17B1 and HSD17B1-associated genes, such as ESR1, ESRRA, PPARG, AKT1, and AKR1C1\2\3, were involved in several estrogen metabolism pathways, such as steroid binding, 17-beta-hydroxysteroid dehydrogenase (NADP+) activity, steroid hormone biosynthesis, and regulation of hormone levels. The molecular basis of the effects of kaempferol treatment was evaluated.

**Conclusions:**

Kaempferol is a novel therapeutic candidate for EC via HSD17B1-related estrogen metabolism pathways. These results provide new insights into the efficiency of the medical translation of phytoestrogens.

**Supplementary Information:**

The online version contains supplementary material available at 10.1186/s12967-023-04048-z.

## Introduction

Endometrial cancer (EC) is the most prevalent gynecologic cancer worldwide, and the rising incidence of EC makes it an important concern for women's health, particularly in industrialized nations [1, 2]. Although it is relatively easily treatable in the early stages, treatments for advanced and recurrent EC are rarely therapeutic and have a poor prognosis [3]. Medroxyprogesterone acetate (MPA) at high doses has been licensed for the treatment of EC. However, this progestin treatment is ineffective in up to 30% of individuals with endometrial hyperplasia and endometrioid cancer [4]. Recently, molecularly targeted therapy has also made progress, but there are still few acceptable treatments [5]. Therefore, the development of new, potent therapies against EC remains urgently necessary.

Kaempferol is a flavonol that is commonly found in plants, such as tea, brassicas, legumes, and certain fruits, and constitutes a significant part of the human diet [6]. Several studies have revealed that kaempferol is substantially less toxic to normal cells than standard chemotherapeutic drugs [7]. Recent studies have suggested that kaempferol exerts anti-cancer effects in hormone-related cancers in vitro, likely through interaction with the estrogen receptors (ERs) [8–11]. The majority of the studies were conducted using in vitro methods such as the MTT assay, brine shrimp lethality (BSL) bioassay, and sulforhodamine B (SRB) assay, etc. More studies are required to determine whether kaempferol is associated with anti-cancer properties. Furthermore, in vivo and in silico studies are needed to thoroughly investigate the relationship among potential targets and associated pathways of kaempferol in EC, particularly hormone-related EC, for the discovery of new anti-cancer drugs. Thus, further study is needed to comprehensively evaluate the effects and toxicity of kaempferol on EC cells in vitro and in vivo, to understand its potential underlying molecular mechanisms of action, and to evaluate its potential as a therapeutic agent for EC.

The network pharmacology-based strategy is a developing discipline that elucidates its underlying multi-component, multi-target, and multi-pathway mode of action against different diseases, especially cancer [12]. Meanwhile, network pharmacology constructs a multi-level network and methodically explores the connection between potential active compounds and EC from an overall perspective. Then, the probable active compounds and target proteins were further screened in a molecular docking approach that defines the behavior of small molecules in the binding site of proteins and seeks to clarify essential biochemical processes [13]. Integrating the above approach with experimental verification has been widely used to discover potential drugs [14–16].

In this study, experimental studies including in vitro, in vivo and transcriptomic sequencing proved the regulation of targets by kaempferol in the effects and toxicity. Then, we integrated system pharmacology and molecular docking with experimental verification to clarify the underlying mechanisms of kaempferol for the treatment of EC. The target-pathway network was constructed through the data obtained above. Furthermore, GO analysis and KEGG pathway enrichment analysis were applied to analyze the anti-cancer molecular mechanism of kaempferol. Finally, the potential mechanisms of kaempferol were validated.

## Materials and methods

### Culturing cells and evaluating cell viability

Human EC cell lines (HEC-1-A, HEC-1-B, KLE, and AN3 CA cells), human lung fibroblast cell line (MRC-5), human hepatic cell line (WRL 68), and human colon epithelial cell line (CCD 841 CoN)  were derived from the American Type Culture Collection (ATCC, Manassas, VA, USA), while the Ishikawa cell line was derived from the European Collection of Authenticated Cell Cultures (ECACC, Porton Down, Salisbury, UK). Human monocytes were extracted from freshly obtained peripheral venous blood by using the Percoll and Ficoll density gradient procedure [17]. Peripheral venous blood from healthy volunteers was collected after obtaining informed consent according to a protocol approved by the ethical committee of Fujian Maternity and Child Health Hospital (No. 2019-137) and performed in accordance with the Declaration of Helsinki. HEC-1-A and HEC-1-B were maintained in high-glucose Dulbecco’s modified Eagle’s medium (DMEM; Gibco, Carlsbad, CA). KLE, WRL 68, and human monocytes were maintained in Roswell Park Memorial Institute 1640 medium (RPMI-1640; HyClone, Logan, UT). AN3 CA, MRC-5, and CCD 841 CoN were maintained in Minimum Essential Medium (MEM; HyClone). Ishikawa was maintained in a 1:1 mixture (*v*: *v*) of Dulbecco’s modified Eagle’s medium with Ham’s F-12 Nutrient Medium (DMEM/F12; Gibco). All of the mediums were supplemented with 10% fetal bovine serum (Gibco), 1 × 10^5^ units L^−1^ penicillin, and 100 mg L^−1^ streptomycin (Invitrogen, Carlsbad, CA). The cells were incubated in a humidified atmosphere containing 5% CO_2_ at 37 °C. Prior to usage, all cell lines were determined to be mycoplasma-free and were confirmed via short tandem repeat DNA profiling [18].

The effect of kaempferol (Huike Botanical Development, Shanxi, China) on cell viability was determined by MTT or Cell Counting Kit-8 (CCK-8) assays. Briefly, cells were seeded at (1 ~ 3) × 10^4^ cells per well in 200 μL of complete culture medium. On the next day, the medium was replaced with phenol-red-free medium containing 1% serum replacement-2 (Gibco), and the cells were cultured for an additional 24 h. Subsequently, kaempferol (2, 5, 10, 20, and 50 μg mL^−1^) and negative control [NC, 0.5% (*v*: *v*) DMSO], or positive control (2 μg mL^−1^ DDP) were added to the cells. Cell viability was assessed following incubation for a predetermined amount of time (24, 48, or 72 h) at 37 °C in a humidified incubator. MTT (5 mg mL^−1^ in PBS; Sigma-Aldrich, Shanghai, China) was added to each well. After incubation for 4 h, the MTT solution was carefully removed by aspiration, and formazan crystals were dissolved by shaking with 100 μL of DMSO for 30 min. Absorbance was measured at 490 nm. For the human monocytes, MRC-5, WRL 68, and CCD 841 CoN cell lines, kaempferol (2, 5, 10, 20, and 50 μg mL^−1^), negative control [0.5% (*v*: *v*) DMSO] or positive control (2 μg mL^−1^ DDP) was added to the cells. Cell viability was assessed following incubation for 48 h at 37 °C in a humidified incubator. 10 μL of CCK-8 (Genview, Shanghai, China) was added to each well. After incubation for 2 ~ 4 h, absorbance was measured at 450 nm. Each concentration was repeated in three wells, and experiments were repeated three times. Growth inhibition was assessed as the percentage of viable cells, with the viability of negative control (DMSO-treated) cells set to 100%.

### Flow cytometry assay for apoptosis

Apoptotic cells in vitro were detected by flow cytometry, as perviously reported [19]. Briefly, AN3 CA, Ishikawa, HEC-1-A, and HEC-1-B cells were grown to 50 ~ 60% confluence on cell culture slides and were then treated with kaempferol (0, 2, 10, or 50 μg mL^−1^) for 48 h. The cells were then harvested and washed twice with cold PBS (10 mmol L^−1^, pH 7.4), stained with Annexin V-fluorescein isothiocyanate and propidium iodide (PI) in binding buffer (BD Biosciences, San Jose, CA), and detected using the FACSCanto™ II system (BD Biosciences) after 15 min of incubation at room temperature (20 ~ 25 °C) in the dark. Fluorescence was measured at an excitation wavelength of 480 nm through FL-1 (530 nm) and FL-2 (585 nm) filters. Early-stage apoptosis (Annexin V-positive/PI-negative) and late-stage apoptosis (Annexin V-positive/PI-positive) were quantified.

### Flow cytometry assay for cell cycle

PI staining is a commonly used method to analyze the distribution of cells in different phases of the cell cycle. Briefly, AN3 CA cells were grown to 50 ~ 60% confluence on cell culture slides and were then treated with kaempferol (0, 2, 5, 10, or 20 μg mL^−1^) for 48 h. Then, the cells were harvested, washed with cold PBS, and fixed in ice-cold 70% ethanol overnight at 4℃. After fixation, the cells were washed twice with PBS, incubated with PI staining solution (BD Biosciences, San Jose, CA) for 0.5 h at room temperature, and detected using the FACSCanto^™^ II system. The fraction of cells in the G_0_/G_1_, S, and G_2_/M phases of the cell cycle was analyzed using FlowJo software (version 10.0.7, Tree Star Inc., Ashland, OR, USA).

### Colony formation assay

AN3 CA cells were seeded in a 6-well plate with 600 cells per well. Following cell adhesion, the cells were treated with kaempferol at a range of concentrations (0, 0.5, 1, 2, or 5 μg·mL^−1^) or DDP (2 μg·mL^−1^, positive control). For around 2 weeks, colonies were clearly formed. Then, cells were incubated with 4% paraformaldehyde (PFA) for 30 min at 4 °C and stained with a 0.1% (*v*: *v*) crystal violet solution for an additional 30 min. After washing away the crystal violet solution and allowing for air drying, the colonies were photographed under a microscope. The number of colonies with more than 50 cells and colony surface area were quantified by ImageJ software (version 1.51, NIH, U.S.).

### Wound healing assay

AN3 CA or HEC-1-A cells (5 × 10^5^ cells per well) were cultivated in a 6-well plate and allowed to develop to about 80% confluence. The tip of a 10 μL pipette was used to carefully scrape the monolayer of EC cells. Then, the cellular debris was removed using PBS. The specified kaempferol concentrations (0, 2, 10, and 20 μg mL^−1^) were exposed to EC cells. Images of gaps were captured under a microscope when EC cells have been treated with kaempferol for 0, 24  and 48 h, respectively. Bar graphs were created as the widths of the gaps were measured.

### Cell migration assay

The upper side of a 24-well Transwell^®^ chamber (6.5 mm diameter polycarbonate membrane with 8 μm pore size, Costar, Corning, NY, U.S.) was filled with 200 μL AN3 CA or HEC-1-A cells suspensions (1 × 10^5^ cells per well) with specified kaempferol concentrations (0, 2, 10, 20, or 50 μg mL^−1^) in phenol-red-free matching medium containing 12% FBS. The lower chambers were filled with 1000 μL phenol red-free matching medium with 12% FBS. Following 24 h of incubation in 5% CO_2_ at 37 °C, cells that were still located on the upper side of the membrane were wiped with cotton swabs. Cells on the lower side of slide were stained with 0.1% crystal violet solution and then photographed under a microscope. The number of migrated cells was counted in five random fields by ImageJ software.

### Cell invasion assay

The inhibitory potential of kaempferol against the invasion of AN3 CA or HEC-1-A cells was evaluated by a cell invasion assay. The upper chambers of the 24-well Transwells^®^ (6.5 mm diameter polycarbonate membrane with 8 μm pore size) were covered with 50 μL 10% (*v:v*) Matrigel^®^ (Corning, NY, U.S.) and solidified at 37 ℃ for 0.5 h. The lower chambers were added with 1000 μL of phenol red-free matching medium containing 12% FBS. Cells were placed at a density of 1 × 10^5^ cells per well (200 μL) into the upper chambers in phenol-red-free matching medium with 12% FBS containing specified kaempferol concentrations (0, 2, 10, 20, or 50 μg mL^−1^). The subsequent procedure was similar to the cell migration assay. The number of invasive cells was counted in five random fields by ImageJ software.

### Xenograft animal study

Specific pathogen-free female BALB/c nude mice (4 ~ 6 weeks old) were purchased from Beijing Vital River Laboratory Animal Technology Co., Ltd. (Beijing, China) and were maintained on a 12 h/12 h light/dark cycle in a low-stress environment (22 °C, 50% humidity, and low noise). Mice were fed  *ad libitum* with food and water. A specialist monitored their health status during the study based on body weight, physical appearance, and measurable clinical signs (changes in general appearance, hair, eyes, and nose). No adverse clinical signs were observed prior to the experiment. Suspensions of 1 × 10^7^ cells in 200 μL of serum-free medium were injected subcutaneously into the right flank of each nude mouse. Two weeks after AN3 CA or HEC-1-A cells induction, mice with visible tumors (≥ 5 mm) were randomly assigned to five groups (*n* = 10) according to tumor volume and body weight ranges. Kaempferol [150, 300, and 600 mg kg^−1^ dispersed in poloxamer 188 (Pluronic^®^ F-68, BASF SE, Ludwigshafen, Germany) with a 1:5 (*w*: *w*) ratio] was administered to mice intragastrically each day. Negative control mice were administered the vehicle in the same manner. In addition, DDP (5 mg kg^−1^ prepared in 0.9% normal saline) was administered intraperitoneally once a week as a positive control. Tumor sizes were measured using a caliper every 4 days, and volumes were calculated using the standard formula: V = (length × width × width)/2. Body weights were recorded prior to dosing and weekly thereafter. When the tumor volume in the negative control group reached approximately 1,000 mm^3^, the mice were euthanized. The tumor tissue was carefully excised, weighed, frozen in liquid nitrogen, and stored at −80 °C until analysis. The rate of inhibition (IR) was calculated according to the following formula: IR (%) = (1 − mean tumor weight of the experimental group/mean tumor weight of the negative control group) × 100. Organ coefficients were calculated according to the following formula: organ coefficient (%) = (mean weight of the organ/mean mouse body weight) × 100. Brain coefficients were calculated according to the following formula: brain coefficient (%) = (mean weight of the organ/mean weight of the brain) × 100. No animals were excluded from the experiments. Animal studies were approved by the Fujian Medical University Ethical Committee on Experimental Animal Care and Use (No. 2016-13) and were performed in accordance with institutional and national guidelines.

### Cell death assay

The terminal deoxynucleotidyl transferase-mediated dUTP nick-end labeling (TUNEL) assay was conducted using tumor tissues to evaluate apoptosis in situ. Previously sectioned and fixed tumor samples were processed using the In Situ Cell Death Detection Kit (Roche Diagnostics, Basel, Switzerland) according to the manufacturer’s protocol. To determine TUNEL expression in tissue sections, apoptotic events were counted in ten random fields by confocal laser scanning microscopy (TCS SP2; Leica, Wetzlar, Germany). The apoptotic rate of cancer cells (expressed as a percentage) was calculated as apoptotic cells/total cells × 100. The effect of serial concentrations of kaempferol on apoptosis was assessed as the percentage of apoptotic cells in which the control cells were considered 100% viable.

### Next-generation RNA sequencing and bioinformatics analysis

Next-generation RNA sequencing analysis was performed by BioTechnology Co. (Shanghai, China). Briefly, a density of 1 × 10^7^ EC cells·mL^−1^ (HEC-1-A and Ishikawa) was seeded onto 25 cm^2^ cell culture flasks (Corning, Lowell, CA) and then treated with 0.5% DMSO (negative control) or kaempferol (10 μg mL^−1^) for 48 h. Total RNA was extracted with TRIzol Reagent (Life Technologies, Carlsbad, CA) following the manufacturer’s protocol, and the RNA integrity number was determined using an Agilent Bioanalyzer 2100 (Agilent Technologies, Santa Clara, CA). The total RNA was further purified using the RNAClean XP Kit (Beckman Coulter, Inc., Brea, CA) and the RNase-Free DNase Set (QIAGEN, Hilden, Germany). Libraries were constructed (TruSeq RNA Library Prep Kit) and sequenced (Illumina HiSeq 2500) with Vanderbilt VANTAGE. Raw reads were aligned to the human reference genome hg38 (Genome Reference Consortium GRCh38), and differential gene expression was analyzed using CLC Workbench 10.0. Genes with absolute fold change values of  > 2.5 and false discovery rates of  < 0.05 were considered significantly differentially expressed. Hierarchical clustering of negative control (0.5% DMSO) or kaempferol-treated (10 μg mL^−1^) cells was conducted by one minus Spearman’s rank correlation coefficient and total linkage.

### Predicting prospective targets for kaempferol during EC treatment

In brief, unique predicted targets of kaempferol were obtained from the TCMSP, Therapeutic Target Database (TTD, http://db.idrblab.net/ttd/), STITCH 5.0 (http://stitch.embl.de/), DrugBank (https://go.drugbank.com/), ConsensusPathDB (CPDB, http://cpdb.molgen.mpg.de/CPDB), PharmMapper (http://www.lilab-ecust.cn/pharmmapper/), SwissTargetPrediction (http://www.swisstargetprediction.ch/) and Similarity ensemble approach (SEA, https://sea.bkslab.org/) [20–27]. Disease-related targets were identified using the keywords “endometrial cancer OR endometrial carcinoma” from four databases as follows: The Online Mendelian Inheritance in Man (OMIM) database (https://www.omim.org/), the GeneCards database (https://www.genecards.org/), the Gene Expression Profiling Interactive Analysis 2(GEPIA2, https://portal.gdc.cancer.gov/), and the Therapeutic Target Database (TTD) (http://db.idrblab.net/ttd) [^22^, ^28^, ^29^, ^30^]. GEPIA2 database analyzes the RNA sequencing expression data of cancers and normal samples from the Cancer Genome Atlas (TCGA) and the Genotype-Tissue Expression (GTEx) projects with a standard processing pipeline [28]. TTD is a database that contains details on the known and explored therapeutic protein and nucleic acid targets, the targeted disease, the pathway, and the matching pharmaceuticals that are directed at each of these targets [22]. Prior to the intersection, the protein names of all targets were normalized into corresponding standard gene names via UniProt (http://www.uniprot.org/). The intersection of genes between kaempferol’s potential target and endometrial cancer-related genes was identified and visualized with the online Venn’s diagram tool Venny 2.1 (http://bioinfogp.cnb.csic.es/tools/venny/), which were considered as potential pharmacological targets of kaempferol for EC treatment.

### Constructing and analyzing a network-based compound-disease target interaction

The unique genes with potential targets for the EC and kaempferol were matched to construct a kaempferol-disease target database. The kaempferol and the putative targets were loaded into Cytoscape software (version 3.9.1) to establish a network-based interaction [31].

### Protein-protein interaction (PPI) analyzing core targets by the Retrieval of Interacting Genes/Proteins (STRING)

The gene names of intersection targets were imported into the STRING (version 11.5) database (https://string-db.org/, accessed on October 6, 2022) to fully explore the potential mechanism by which KG treats EC by PPI network [32]. The organism was set as Homo sapiens, and the minimum required interaction score was set as 0.7. The Cytoscape plugin Molecular Complex Detection (MCODE, version 2.0.2) was utilized to identify densely connected network modules (or clusters) [33]. Those highly-linked core targets were discovered using the Cytoscape plugin cytoHubba (version 0.1) by the maximal clique centrality (MCC) method, which is superior to predicting crucial proteins from the PPI network [34].

### Enrichment analyzing core targets by GO and KEGG

GO enrichment analysis was performed to categorize the core targets into sections on cellular component (CC), biological process (BP), and molecular function (MF). Key pathways were identified using KEGG, a database for systematic analysis of gene function, with a criterion of *P* < 0.05, and then candidate targets were chosen based on clinical and pathological data for further research [35]. A powerful visualization Cytoscape plugin ClueGO (version 2.5.9), was used to explore and visualize the potential KEGG pathways and GO functions of the core targets [36].

### Overall survival (OS) of core genes expression in EC patients

The OS data from TCGA and the GTEx projects was searched on October 6, 2022, to evaluate the expression level and overall survival (OS) of core genes in normal and EC tissues in the GEPIA2 database [28]. For the OS analysis, patients with EC were divided into cohorts with high and low expression. A Kaplan–Meier survival plot was used to compare the two cohorts, and the hazard ratio (HR) with 95% confidence intervals and the log-rank *P* value were computed. The Human Protein Atlas (HPA) (https://www.proteinatlas.org/) database was used to analyze the immunohistochemical data on the expression of the histone family proteins in EC in order to investigate the protein level expression of these core genes [37].

### Analyzing potential targets by molecular docking

Molecular docking was conducted to evaluate the strength and mechanism of interactions between the candidate targets and active chemicals at the molecular level. We obtained the active compounds' chemical structure from the PubChem database (http://pubchem.ncbi.nlm.nih.gov) and the PDB-formatted 3D crystal structures of the core targets from the RCSB Protein Data Bank (PDB, https://www.rcsb.org/). The binding cavity was searched in CB-Dock2 (https://cadd.labshare.cn/cb-dock2/php/index.php) by improved blind docking [38, 39]. The degree value of binding cavities was generated in accordance with CB-Dock2 using a grid of 50 × 50 × 50 points with a grid spacing of 0.375 Å. AutoDock Vina (version 1.5.6) evaluated the binding activity between the components and the targets based on the magnitude of the binding energy. The top-ranked docking parameters were predicted using affinity and score values. The conformations with the lowest docking scores (highest affinity) were mapped and visualized via PyMOL software (version 2.3.0, Schrödinger, LLC, New York, NY, U. S. ).

### Extracting Total RNA and Real-Time Quantitative PCR (RT-qPCR) analysis

Human EC cells (HEC-1-A, AN3 CA, and KLE) were treated with kaempferol (0, 10, or 50 μg mL^−1^ in DMSO). After 48 h, the cells were collected. The TRIzol (Invitrogen) was utilized for total RNA extraction in accordance with the manufacturer's instructions. A total of 2 μg RNA was reverse transcribed to cDNA using GoScript^™^ Reverse Transcription Mix, Oligo (dT) Kit (Promega, Shanghai, China). RT-qPCR reactions were performed in accordance with the manufacturer’s instructions using Eastep^™^ qPCR Master Mix Kit (Promega) on a LightCyler^®^ Z480 (Roche, Basel, Switzerland). The primers for RT-qPCR were used in the following sequence: HSD17B1 (98 bp), forward (F): 5’-GTGCTGGTGTGTAACGCAG-3’, reverse (R): 5’-GTCCCTACTACATTCACGTCCA-3’; CYP1A1 (141 bp), F: 5’-TCGGCCACGGAGTTTCTTC-3’, R: 5’-GGTCAGCATGTGCCCAATCA-3’; CYP1B1 (197 bp), F: 5’-TGAGTGCCGTGTGTTTCGG-3’, R: 5’-GTTGCTGAAGTTGCGGTTGAG-3’; ESRRA (100 bp), F: 5’-ACCGAGAGATTGTGGTCACCA-3’, R: 5’-CATCCACAGCTCTGCAGTACT-3’; PPARG (107 bp), F: 5’-GAAGTTCAATGCACTGGAATTAGATG-3’, R: 5’-CCTCGATGGGCTTCACGTT-3’; AKT1 (96 bp), F: 5’-AGCGACGTGGCTATTGTGAAG-3’, R: 5’-GCCATCATTCTTGAGGAGGAAGT-3’; AKR1C1/2/3 (108 bp), F: 5’-GTCATCCGTATTTCAACCGGAG-3’, R: 5’-CCACCCATCGTTTGTCTCGTT-3’; GAPDH (138 bp), F: 5’-GCACCGTCAAGGCTGAGAAC-3’, R: 5’-TGGTGAAGACGCCAGTGGA-3’. These qPCR reactions utilized a standard protocol that included 40 cycles and 10 s of annealing at 60 ℃. The relative expression of the target genes was calculated and normalized using the 2^−ΔΔCt^ method.

### Protein extraction and western blotting

Human EC cells (HEC-1-A, AN3 CA, and KLE) were treated with kaempferol (0, 10, or 50 μg mL^−1^ in DMSO). After 48 h, the cells were collected and washed with cold PBS (10 mmol L^−1^, pH 7.4), incubated in ice-cold mammalian protein extraction reagent according to the manufacturer’s protocol (Thermo Fisher Scientific, Waltham, MA), and placed on ice for 0.5 h. The supernatant after centrifugation at 15,000 × *g* for 15 min at 4 °C was collected. Tumor tissues were homogenized in tissue protein extraction reagent according to the manufacturer’s protocol (Thermo Fisher Scientific). After incubation at 4 °C for 0.5 h, the homogenate was centrifuged at 16,000 × *g* at 4 °C for 0.33 h, and the supernatant was collected. The protein concentration in the supernatant was quantified by Bio-Rad Protein Assay Reagent (Bio-Rad Laboratories, Hercules, CA) according to the manufacturer’s protocol. The whole cell lysates and tumor homogenates (50 μg) were resolved on an 8 ~ 12% SDS–polyacrylamide gel, transferred to a polyvinylidene difluoride membrane (NEN Life Sciences, Boston, MA), probed sequentially with antibodies against ESR1 (ab108398, 67 kDa), ESRRA (ab137489, 55 kDa), PPARGC1A (ab188102, 91 kDa) (Abcam, Cambridge, MA, U. S.), CASP3/p17/p19 (19677–1, 35 kDa), CASP9/p35/p10 (66169–1, 46 kDa), PPARG (16643–1, 55 kDa), HSD17B1 (25334–1, 35 kDa) (Proteintech, Wuhan, China) at 4 °C overnight, rinsed, and incubated with the goat anti-rabbit secondary antibody (Abcam, Cambridge, MA). Immunoreactive bands were visualized using the electrogenerated chemiluminescence (ECL) Detection System (Pierce, Rockford, IL). ACTB (β-actin, Sigma) was used as the loading control, and protein levels were normalized to its levels.

### Hematoxylin and eosin (HE) staining and immunohistochemical analysis (IHC)

Paraffin Sects. (5 μm) were used for histological analysis. Tumor sections were subjected to HE staining and viewed under an optical microscope (magnification: × 200; Leica). Images were captured with a Leica digital camera. The sections were deparaffinized with xylene followed by a descending series of ethanol concentrations. Antigen retrieval was carried out in a microwave-heated citrate buffer (pH 6.0) for 0.33 h. Endogenous peroxidases were blocked with 3% H_2_O_2_/methanol at room temperature for 0.25 h. Non-specific epitopes were blocked with 1% normal goat serum at room temperature for 0.5 h. The tumor sections were incubated with antibodies at 4 °C overnight. The immunoreactions were visualized using a streptavidin-biotin complex method followed by a diaminobenzidine reaction (Zymed, South San Francisco, CA). The tumor sections were counterstained with hematoxylin to visualize the nuclei. The immunoreactions were viewed under an optical microscope (magnification: × 400; Leica), and images were obtained using a digital camera (Leica).

### Statistical analysis

All results are expressed as means ± standard errors (SEs). Statistical comparisons were performed using the *t*-test and one- or two-way analysis of variance (ANOVA) with Turkey’s *post-hoc* tests. Statistical analyses were implemented in GraphPad Prism 8.0 Software (GraphPad, Inc., La Jolla, CA, U.S.). *P* values less than 0.05 (*), 0.01 (**), and 0.001 (***) were considered statistically significant.

## Results

### Kaempferol inhibited proliferation abilities among EC cells with different ER subtypes

**Fig. 1:**
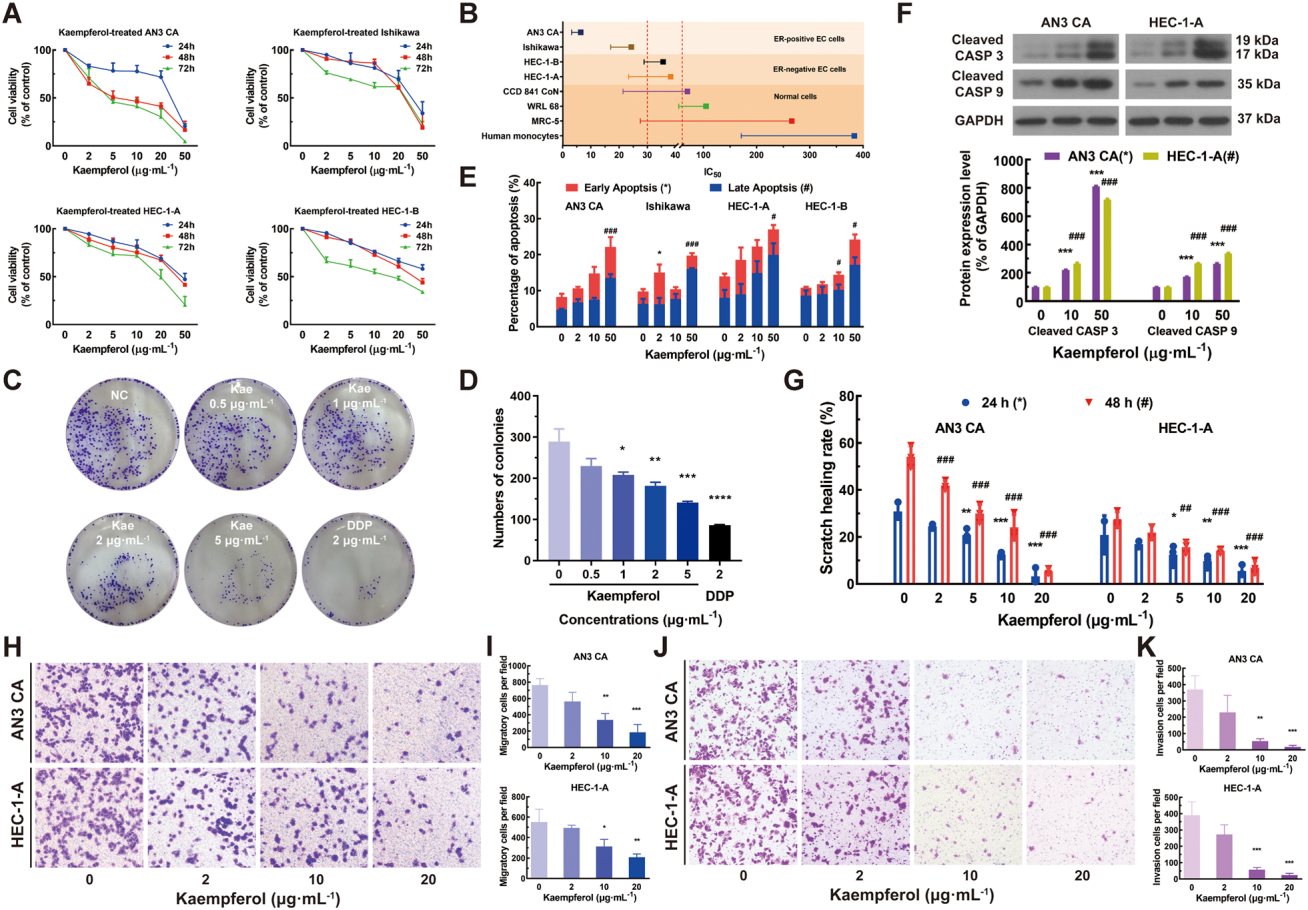
Kaempferol suppressed the proliferation, promoted apoptosis, and limited the tumor-forming, invasion, and migration capacities of both ER-subtype human endometrial cancer cells. **A** Kaempferol inhibited human endometrial cancer cells' proliferation (ER-positive AN3 CA and Ishikawa, ER-negative HEC-1-A and HEC-1-B EC cells) in a dose- and time-dependent manner. **B** The IC50 values of kaempferol at 48 h were 6.38, 24.35, 35.62, 38.31, 69.87, 104.90, 266.10, and 383.60 μg·mL^−1^ for AN3 CA, Ishikawa, HEC-1-A, HEC-1-B, CCD 841 CoN, WRL 68, MRC-5 and human monocytes, respectively. **C**–**D** Kaempferol significantly inhibited AN3 CA cell colony formation in a dose-dependent manner. **E** The early apoptotic cells and late-stage apoptotic cells of EC cells (AN3 CA, Ishikawa, HEC-1-A, and HEC-1-B) were both increased after treatment with kaempferol in a dose-dependent manner. **F** Kaempferol induced cell apoptosis by elevating the expression of cleaved CASP3 and cleaved CASP9 in a dose-dependent manner. **G** Kaempferol inhibited EC cells’ wound healing in a time- and dose-dependent manner. In a concentration-dependent manner, kaempferol inhibited migration **H**–**I** and invasion **J**–**K**. The results are presented as means and standard deviations (SDs) from triplicate experiments. Kae: Kaempferol; Compared with the negative control, ^*, #^*P* < 0.05, ^**, ##^*P* < 0.01, ^***, ###^*P* < 0.001

EC is a heterogeneous disease that can contain a mixture of ER-positive and ER-negative cancer cells. In order to evaluate the efficacy of kaempferol in treating EC, the effect of kaempferol on the proliferation of different ER subtypes of EC cell lines was studied, including AN3 CA (ER-positive, PTEN nonsense mutation), Ishikawa (ER-positive, PTEN inactive mutation), HEC-1-A (ER-negative, wild-type PTEN), and HEC-1-B (ER-negative, wild-type PTEN)[40–46]. The EC cells were treated with increasing doses of kaempferol (0, 2, 5, 10, 20, and 50 μg mL^−1^) for 24, 48, or 72 h. We observed a dose- and time-response relationship when the cells were treated with kaempferol (Fig. 1A). The rate of inhibition stabilized at nearly 80% after 48 h, and thus 48 h of treatment was selected for subsequent analyses. For the ER-positive EC subtypes AN3 CA and Ishikawa, the IC_50_ values of kaempferol at 48 h were 6.38 and 24.35 μg mL^−1^, while for the ER-negative EC subtypes HEC-1-A and HEC-1-B, they were 35.62 and 38.31 μg mL^−1^, respectively (Fig. 1B). Moreover, the ER-positive and ER-negative subtypes of EC cell lines showed different responses to treatment with DDP at a concentration of 2 μg mL^−1^ for 48 h (Additional file 1: Fig. S1A). The ER-positive cells had significantly lower percentages of cell viability compared to the ER-negative cells, with values of 44.99% and 39.49% for AN3 CA and Ishikawa, respectively, compared to 70.73% and 66.02% for HEC-1-A and HEC-1-B, respectively (Additional file 1: Fig. S1A, *P* < 0.001). These findings demonstrated that kaempferol can inhibit EC cell proliferation, regardless of ER subtype. However, the extent of inhibition may vary based on different ER subtypes of EC cell lines.

### Kaempferol inhibited AN3 CA cell colony formation

We further evaluated the colony-forming potential of kaempferol in AN3 CA cells using the clonogenic assay, which measured the capacity of a single cell to grow into a colony of at least 50 cells. Kaempferol inhibited AN3 CA cell colony formation in a dose-dependent manner (Figs. 1C–D). Specifically, colony formation was significantly reduced in AN3 CA cells treated with kaempferol at concentrations ranging from 1 to 5 μg mL^−1^ when compared with the NC (*P* < 0.05). These findings suggested that kaempferol had the potential as a therapeutic agent for endometrial cancer by inhibiting the colony formation ability of cancer cells.

### No significant toxicity of kaempferol was found in human monocytes and normal cell lines at non-cytotoxic concentrations

In order to determine the safety of kaempferol for use as a potential therapeutic agent, its effect on human non-transformed monocytes or normal cell lines was evaluated. The study utilized a CCK-8 assay on peripheral-venous-blood extracted human monocytes, MRC-5, WRL 68, and CCD 841 CoN cell lines incubated with increasing doses of kaempferol (0, 2, 5, 10, 20, and 50 μg mL^−1^) for 48 h. Kaempferol had low cytotoxicity towards these healthy cells, with IC_50_ concentrations ranging from 69.87 μg mL^−1^ to 383.6 μg mL^−1^ (Additional file 1: Fig. S1B–E and Fig. 1B). Additionally, the human healthy cells had significantly higher percentages of cell viability compared to the ER-positive cells in treatment with DDP at a concentration of 2 μg mL^−1^ for 48 h, with values of 71.02%, 66.63%, 51.70%, and 75.53% for MRC-5, CCD 841 CoN, WRL 68, and monocytes, respectively (Additional file 1: Fig. S1A, *P* < 0.05).

### Kaempferol induced apoptosis in EC cells with re-distribution of the cell cycle

To determine whether the kaempferol-induced growth inhibition of EC cells was related to apoptosis, Annexin-V/PI staining was performed to evaluate pro-apoptotic activity. After treatment with kaempferol at a concentration of 2 μg mL^−1^ for 48 h, the percentage of early apoptotic cells increased in the ER-positive Ishikawa cell line (*P* < 0.05, Fig. 1E and Additional file 2: Fig. S2A), and after treatment with kaempferol at a concentration of 50 μg mL^−1^ for 48 h, the percentage of late apoptotic cells increased in all cell lines (*P* < 0.05, Fig. 1E and Additional file 2: Fig. S2A). Histograms displayed the percentages of early apoptosis, late apoptosis, and total apoptotic cells after 48 h treatment (Fig. 1E). Kaempferol induced cell apoptosis by elevating the expression of cleaved CASP3 and cleaved CASP9 in a dose-dependent manner (Fig. 1F). To further investigate the effect of kaempferol on apoptosis, we performed PI staining to analyze the cell cycle distribution of AN3 CA cells. Results showed that the percentage of cells in the S phase increased within the kaempferol concentration range of 2-10 μg mL^−1^, whereas the proportion of cells in the G_0_/G_1_ phase decreased (Additional file 2: Fig. S2B). Additionally, a significant prolongation of the G_2_/M phase was observed at a concentration of 20 μg mL^−1^ (Additional file 2: Fig. S2B). The results suggested that kaempferol had a significant effect on the cell cycle of EC cells, inducing alterations in both the G_0_/G_1_ and S phases, as well as causing cell cycle arrest in the G2/M phase at higher concentrations.

### Kaempferol inhibited scratch healing, migration, and invasion abilities among EC cells with different ER subtypes

The ability of cancer cells to migrate and invade had been hypothesized to play an important role in cancer cell development and metastatic potential, and compounds that can inhibit these processes were potential candidates for cancer therapy. We performed wound healing, migration, and invasion assays to assess the effect of kaempferol on the motility of EC cells *n vitro*. The results revealed that kaempferol inhibited wound healing, migration, and invasion of EC cells in a dose- and time-dependent manner (Figs. 1G–K). At concentrations above 5 μg mL^−1^, kaempferol significantly hindered the healing of EC cells of different ER subtypes, regardless of 24 h and 48 h of exposure. Interestingly, unlike ER-negative HEC-1-A cells, ER-positive AN3 CA cells significantly reduced the scratch healing rate at 2 μg mL^−1^ for 48 h of exposure (Fig. 1G). In addition, kaempferol inhibited the migration and invasion of EC cells in a concentration-dependent manner (Figs. 1H–K). At a concentration of more than 10 μg mL^−1^, kaempferol significantly inhibited migration (Figs. 1H–I) and invasion (Figs. 1J–K) abilities of EC cells, regardless of ER subtypes.

### Kaempferol inhibited tumor growth and promoted apoptosis in both ER subtypes EC mouse xenograft models

**Fig. 2:**
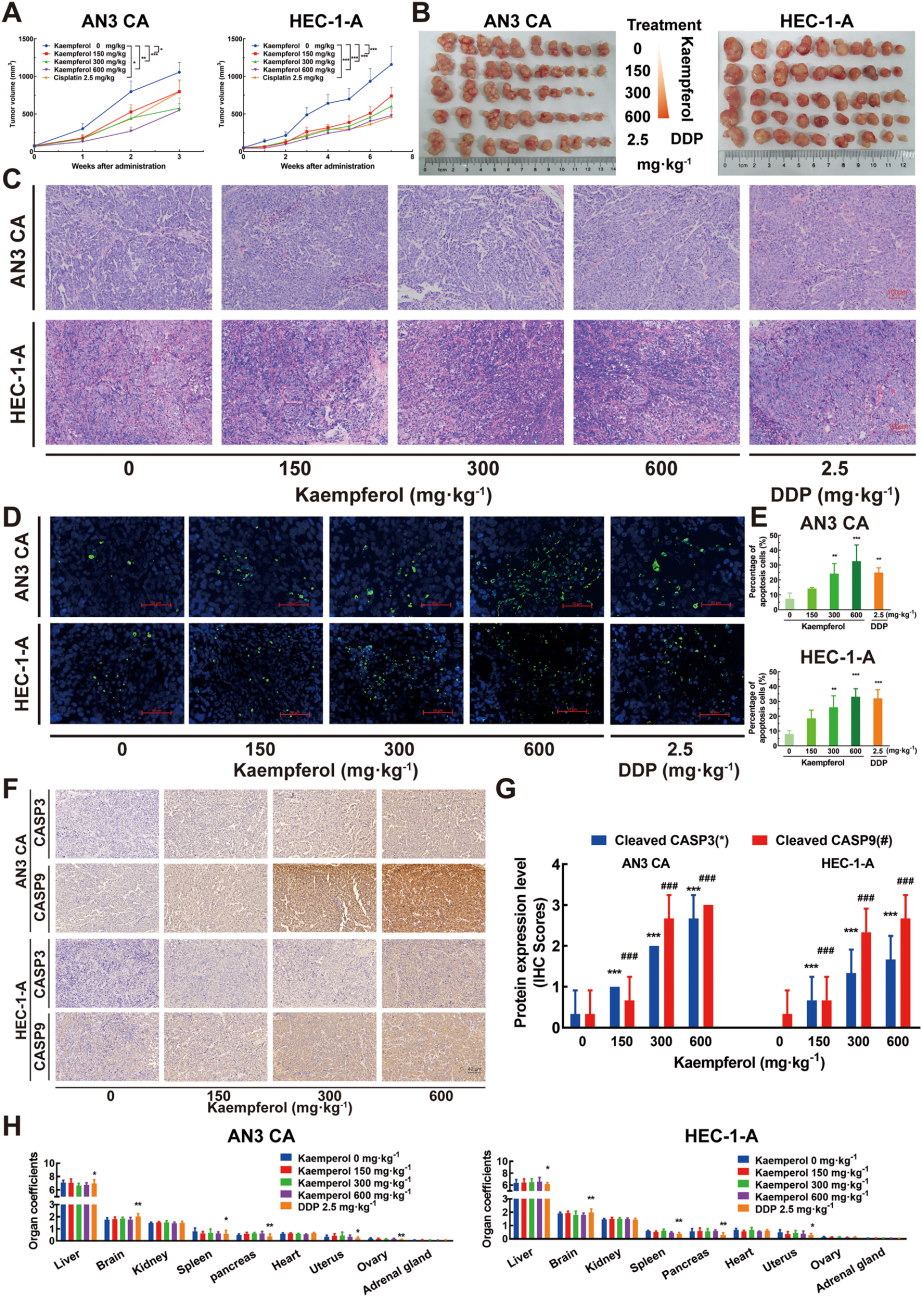
Kaempferol inhibited tumor growth and promoted apoptosis in a human endometrial cancer xenograft mouse model. **A** Kaempferol suppressed the growth of human endometrial cancer cells (AN3 CA and HEC-1-A) in BALB/c nude mice with xenograft tumors in vivo. **B** Macroscopic appearance of tumors after treatment (*n* = 10 per group). **C** After treatment, tumors were excised, weighed, and sectioned at a thickness of 8 μm for hematoxylin and eosin staining. **D** Representative merged images of TUNEL immunofluorescent staining in paraffin sections of tumor tissue of AN3 CA and HEC-1-A xenograft mice treated with vehicle (poloxamer 188; negative control), kaempferol, or DDP. More TUNEL-positive EC cells (AN3 CA and HEC-1-A cells) in tumors from kaempferol-treated mice than in those from negative control mice. Blue represented EC cells, and green represented apoptotic cells. Scale bar = 50 μm. **E** The histograms showed that the percentage of apoptosis cells with the treatment of kaempferol. **F**–**G** With the treatments of kaempferol, cleaved CASP3 and CASP9 increased gradually. **H** No adverse effects or significant changes in organ coefficients was observed in kaempferol-treated and vehicle-treated animals. However, the organ coefficients were significantly changed in several organs in DDP-treated animals compared to those of vehicle-treated animals. Results are presented as means and SDs. Compared with the negative control, ^*, #^*P* < 0.05, ^**, ##^*P* < 0.01, ^***,###^*P* < 0.001

To investigate the effect of kaempferol on the growth of both ER subtypes of EC cells in vivo, female BALB/c nude mice were inoculated with ER-positive subtype AN3 CA and ER-negative subtype HEC-1-A cells to evaluate the effect of kaempferol on tumor growth in xenograft mouse models, using DDP as a positive control. The tumor volume reached 1,000 mm^3^ after 3 weeks (AN3 CA) or 7 weeks (HEC-1-A) in kaempferol-treated mice. Treatment with kaempferol by intragastric administration as well as DDP significantly reduced the tumor volume and suppressed tumor growth in both ER subtypes mouse xenograft models when compared to the vehicle (all *P* < 0.05, Fig. 2A). For AN3 CA cells, the tumor weight IR in the 150 mg kg^−1^, 300 mg kg^−1^, 600 mg kg^−1^ kaempferol-treated, and 2.5 mg kg^−1^ DDP-treated groups was 21.6%, 32.55%, 43.1%, and 29.5%, respectively (Fig. 2B left and Additional file 3: Fig. S3A). For HEC-1-A cells, the tumor weight IR in the 150 mg kg^−1^, 300 mg kg^−1^, 600 mg kg^−1^ kaempferol-treated, and 2.5 mg kg^−1^ DDP-treated groups was 14.3%, 36.4%, 40.9%, and 48.1%, respectively (Fig. 2B right and Additional file 3: Fig. S3B). HE staining was used to assess the effect of kaempferol on the growth and morphology of EC cells in vivo. The results showed that less necrosis occurred in EC tumors treated with kaempferol (Fig. 2C). TUNEL staining of the sections revealed significantly more TUNEL-positive EC cells in tumors from kaempferol-treated mice than in those from negative control mice (Fig. 2D–E). The apoptotic rates of AN3 CA and HEC-1-A cells were significantly increased in response to treatment with 300 mg kg^−1^ and 600 mg kg^−1^ kaempferol, as well as 2.5 mg kg^−1^ DDP, compared to the negative control groups (Fig. 2D–E). Specifically, the apoptotic rates of AN3 CA cells were 24.17 ± 6.81%, 32.60 ± 10.95%, and 25.00 ± 3.16% for the 300 mg kg^−1^, 600 mg·kg^−1^ kaempferol, and 2.5 mg kg^−1^ DDP groups, respectively (Fig. 2D–E top, *P* < 0.05). The apoptotic rates of HEC-1-A cells were 26.08 ± 7.69%, 33.13 ± 5.44%, and 32.00 ± 5.88% for the 300 mg kg^−1^, 600 mg kg^−1^ kaempferol, and 2.5 mg kg^−1^ DDP groups, respectively (Fig. 2D–E bottom, *P* < 0.05). With the treatment of kaempferol, IHC scores of cleaved CASP3 and CASP9 in AN3 CA and HEC-1-A cells were increased gradually (Fig. 2F–G, *P* < 0.05). The results indicated that kaempferol may induce apoptosis in the EC cells by activating the caspase-dependent apoptotic pathway. This was supported by the HE staining and TUNEL assay results, which suggested that the inhibited tumor growth in the kaempferol-treated group could be attributed to increased apoptosis.

### Kaempferol exhibited low toxicity in human EC xenograft models

Systemic toxicity was assessed by monitoring body weight throughout the treatment period and organ coefficients for the main organs excised at the end of the experiment. No adverse effects or significant changes in body weight (Additional file 1: Figs. S3C-D) or organ coefficients (Fig. 2H) were observed in kaempferol-treated and vehicle-treated animals, indicating that kaempferol exhibited little toxicity in mice at the curative dose. However, the organ coefficients were significantly changed in DDP-treated animals in several organs, including the liver, brain, spleen, pancreas, uterus, and ovary, compared to those of vehicle-treated animals in both human EC xenograft models (all *P* < 0.05, Fig. 2H).

### Differentially expressed genes were analyzed in kaempferol-treated EC cells

**Fig. 3:**
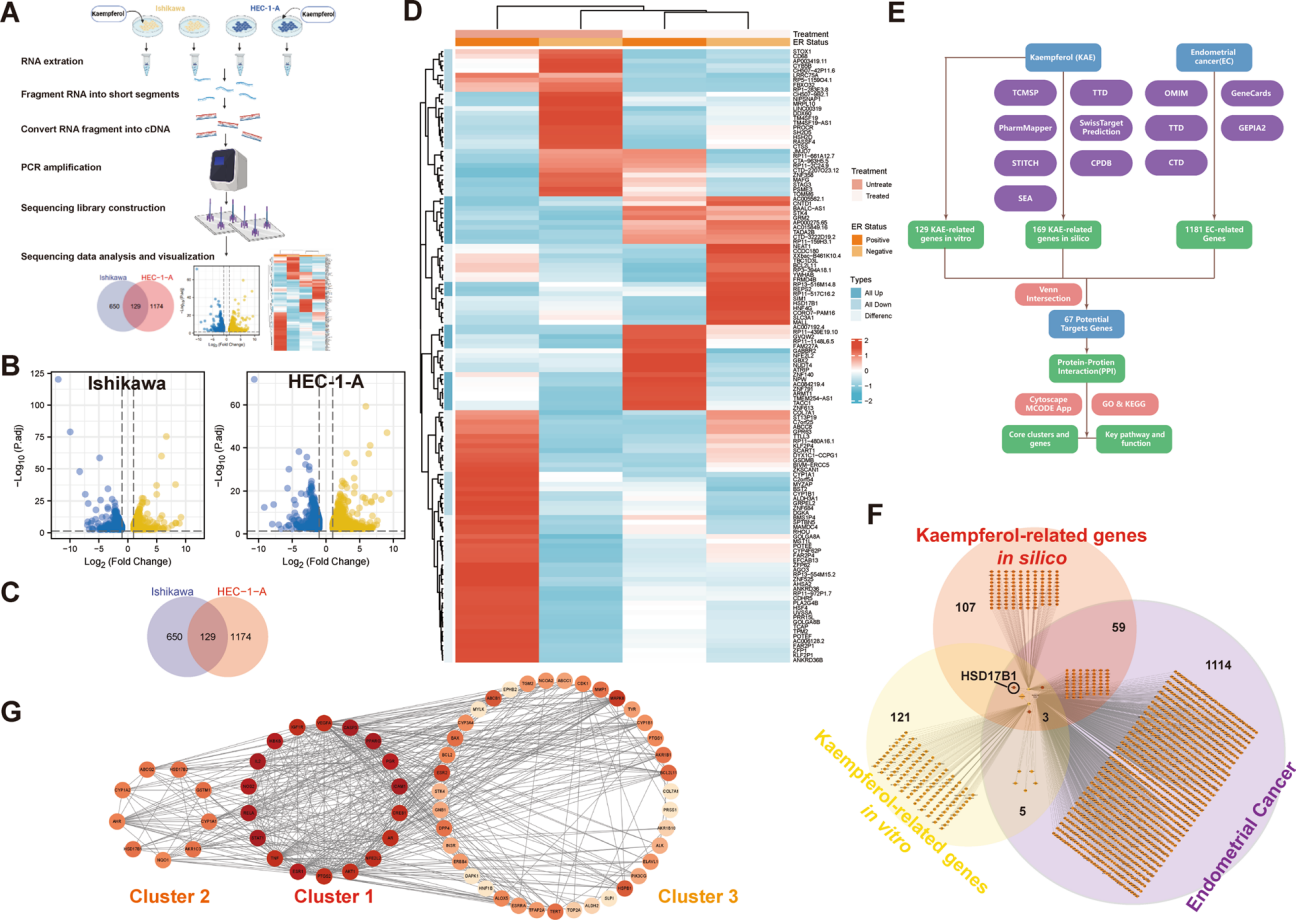
The RNA sequencing and network pharmacology approach identified differentially expressed genes related to overall survival (OS). **A** The strategy of next-generation RNA sequencing in kaempferol-treated and negative control EC cells. **B** Differentially expressed genes (DEGs) between kaempferol-treated and negative control EC cells (Ishikawa and HEC-1-A). Yellow: upregulated differentially expressed genes; Blue: downregulated differentially expressed genes. **C** A total of 129 overlap genes for DEGs between the kaempferol-treated and negative control EC cells of Ishikawa (blue background) and HEC-1-A (red background). **D** Differential types of DEGs were identified between the kaempferol-treated and negative control in Ishikawa (ER-positive) and HEC-1-A (ER-negative) cells. **E** Flowchart for screening endometrial cancer-related genes, kaempferol-related genes, and the related pathway. **F** A total of 3 genes HSD17B1, CYP1B1, and CYP1A1 were identified in three gene sets, including 169 kaempferol-related genes in silico (red background), 129 kaempferol-related genes in vitro (yellow background), and 1181 endometrial cancer-related genes (purple background). **G** 67 potential protein-protein interactions were identified. According to the MCODE score, color brightness indicated the strength of the association, with brighter colors indicating a stronger association

To investigate the potential mechanisms underlying the effects of kaempferol on EC cells, the gene mRNA expression levels in vehicle-treated in comparison with kaempferol-treated ER-positive subtype Ishikawa and ER-negative subtype HEC-1-A cells were assessed using next-generation RNA sequencing (Fig. 3A). Using fold change values of > 2.5 and false discovery rates of < 0.05 as the threshold, a total of 129 differentially expressed genes were identified in both ER subtypes EC cells (Figs. 3B–C). 129 DEGs were identified between the kaempferol-treated and negative control in both Ishikawa (ER-positive) and HEC-1-A (ER-negative) cells (Fig. 3D). These results suggested that kaempferol may exert its anti-tumor effects through the regulation of a variety of molecular pathways involved in cell growth, proliferation, survival, apoptosis, and the immune response.

### Network pharmacology predicted that kaempferol was involved in the anti-cancer effect on EC

We gathered targets for kaempferol from the TCMSP, TTD, STITCH, CPDB, SwissTargetPrediction, PharmMapper, and SEA databases. A total of 169 targets were discovered after integrating UniProt database entries and removing duplicates. EC-related targets were acquired in this study from the OMIM, GeneCards, TCGA, and TTD databases. Specifically, 310 targets with a relevance score of more than or equal to 10 were selected for subsequent analysis in the TCGA database. Additionally, 12 EC-related targets were collected from the OMIM database, and 135 targets were collected from the TTD. A total of 1181 disease target genes were identified after removing duplicates (Fig. 3E). After venn intersection, a total of 3 gene candidates, HSD17B1, CYP1B1, and CYP1A1, were identified in three gene sets, including 169 kaempferol-related genes in silico (red background), 129 kaempferol-related genes in vitro (yellow background), and 1181 endometrial cancer-related genes (purple background) (Fig. 3F). To further investigate the potential pathways of kaempferol, genes that were present in at least two gene sets were considered as potential target genes. Based on this analysis, a total of 67 potential target genes were identified as being involved in the response of EC cells to kaempferol treatment. According to the MCODE score, we identified three potential gene clusters in the PPI network of the DEGs in kaempferol-treated EC cells (Fig. 3G). The color brightness indicated the strength of the association, with brighter colors indicating a stronger association (Fig. 3G). The results showed that candidates for the HSD17B1 and CYP1A1 genes were in cluster 2, and candidate for the CYP1B1 gene were in cluster 3 (Fig. 3G).

### Kaempferol upregulated HSD17B1 expression and sensitivity in ER-negative EC cells

**Fig. 4:**
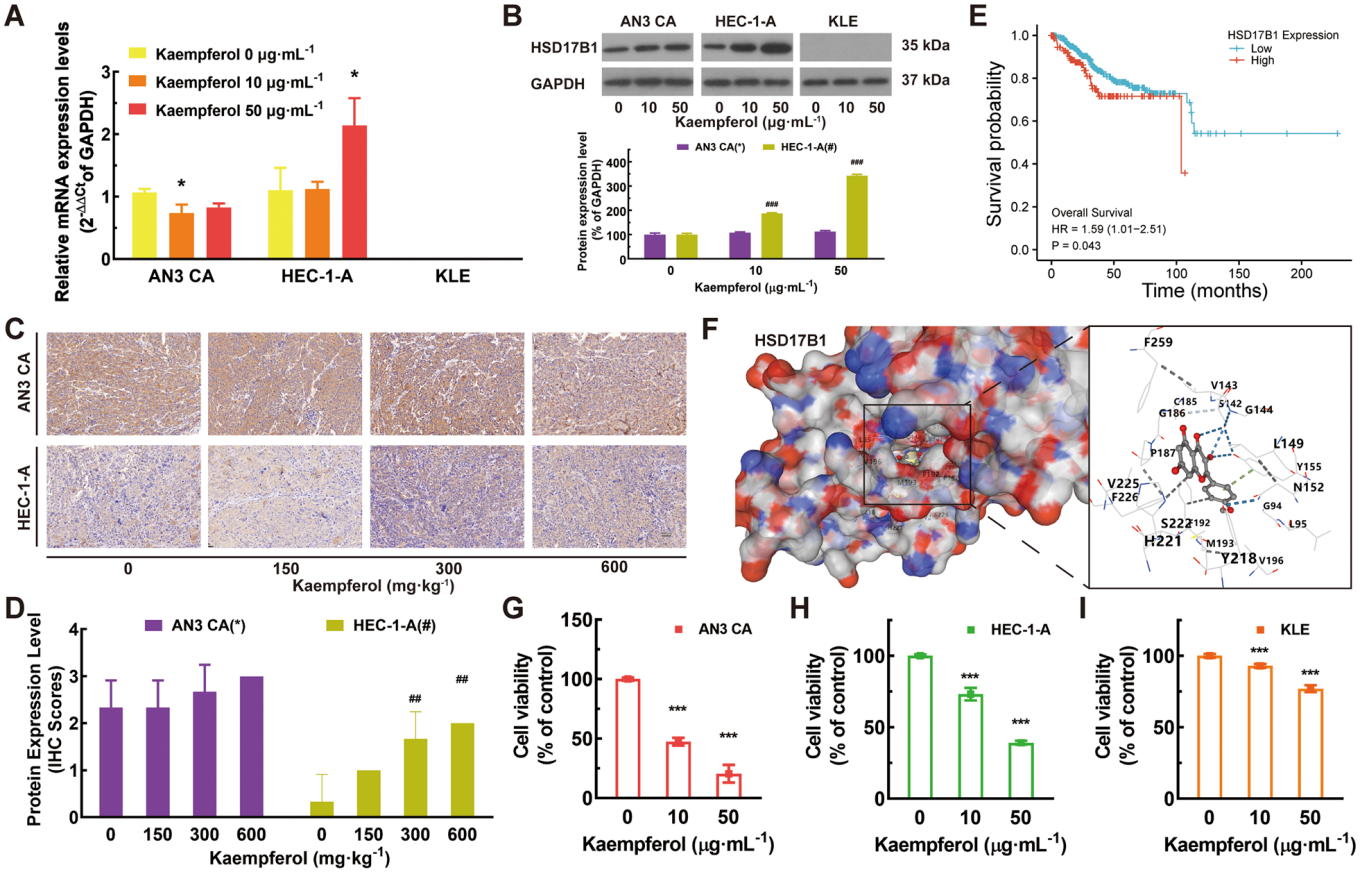
Kaempferol up-regulated HSD17B1 expression and sensitivity in ER-negative EC cells. **A** For the AN3 CA cells, the mRNA expression of HSD17B1 was significantly decreased with 10 μg·mL^−1^ treatment of kaempferol; for the HEC-1-A cells, the mRNA expression of HSD17B1 was significantly increased with 50 μg·mL^−1^ treatment of kaempferol; the mRNA expression of HSD17B1 was barely expressed in KLE cells. **B** The protein expression level of HSD17B1 in ER-positive AN3 CA was unchanged, but the levels of HSD17B1 protein were significantly increased in ER-negative HEC-1-A with kaempferol treatment. And HSD17B1 was barely expressed in KLE cells. **C**–**D** Similar results were also found in the IHC of HSD17B1 in nude mouse tumor tissue after 3–7 weeks of treatment. **E** High expression levels of HSD17B1 had significantly shortened OS in EC patients. **F** Kaempferol may bind to the HSD17B1 hydrophobic LBD through several conserved hydrogen bond interactions with the amino acid residues Y155 and S142. **G** The AN3 CA cells were sensitive to kaempferol with a high level of HSD17B1. **H** Kaempferol can upregulate the expression of HSD17B1 in HEC-1-A. **I** Kaempferol-resistant KLE cells with low HSD17B1 expression

Firstly, the gene expression of gene candidates was verified by RT-qPCR in EC cell lines. CYP1B1 and CYP1A1 were significantly down-regulated in the treatments of kaempferol (Additional file 4: Figs. S4A–B, *P* < 0.01). For AN3 CA cells, the mRNA expression level of HSD17B1 was significantly down-regulated in the 10 μg mL^−1^ treatments of kaempferol (Figs. 4A–B, *P* < 0.05); For HEC-1-A cells, the mRNA and protein expression level of HSD17B1 was significantly up-regulated in the 50 μg mL^−1^ treatments of kaempferol (Figs. 4A–B, *P* < 0.05). Similar results were observed in the IHC of HSD17B1 in nude mouse tumor tissue after several weeks of kaempferol treatment (Figs. 4C–D). We also investigated the clinical relevance of candidate genes using publicly available data from the TCGA database. Furthermore, EC patients with a high HSD17B1 expression level had significantly shortened OS (Fig. 4E), indicating the potential prognostic relevance of HSD17B1 expression in EC. To explore the underlying mechanisms, we conducted further studies of kaempferol and the OS-related hub gene HSD17B1. Molecular docking analysis was performed to obtain insight into the potential binding interactions between kaempferol and the hydrophobic ligand-binding domain (LBD) of HSD17B1. Kaempferol may bind to the HSD17B1 hydrophobic LBD through several conserved hydrogen bond interactions with amino acid residues Y155 and S142 (Fig. 4F). Notably, the high expression level of HSD17B1 was found to be positively correlated with kaempferol sensitivity (Figs. 4G–I). Moreover, kaempferol was found to up-regulate the expression of HSD17B1 in HEC-1-A cells (Fig. 4H), implying that the observed increase in HSD17B1 expression with kaempferol treatment may be due to the direct effect of kaempferol on HSD17B1 expression. Additionally, kaempferol-resistant KLE cells with negligible HSD17B1 expression were identified (Fig. 4I).

### Kaempferol, an ESRRA inhibitor, affected multiple estrogen metabolism pathways and differentially regulated PPARG expression in EC cells of different ER subtypes, independent of its effect on ESR1

**Fig. 5:**
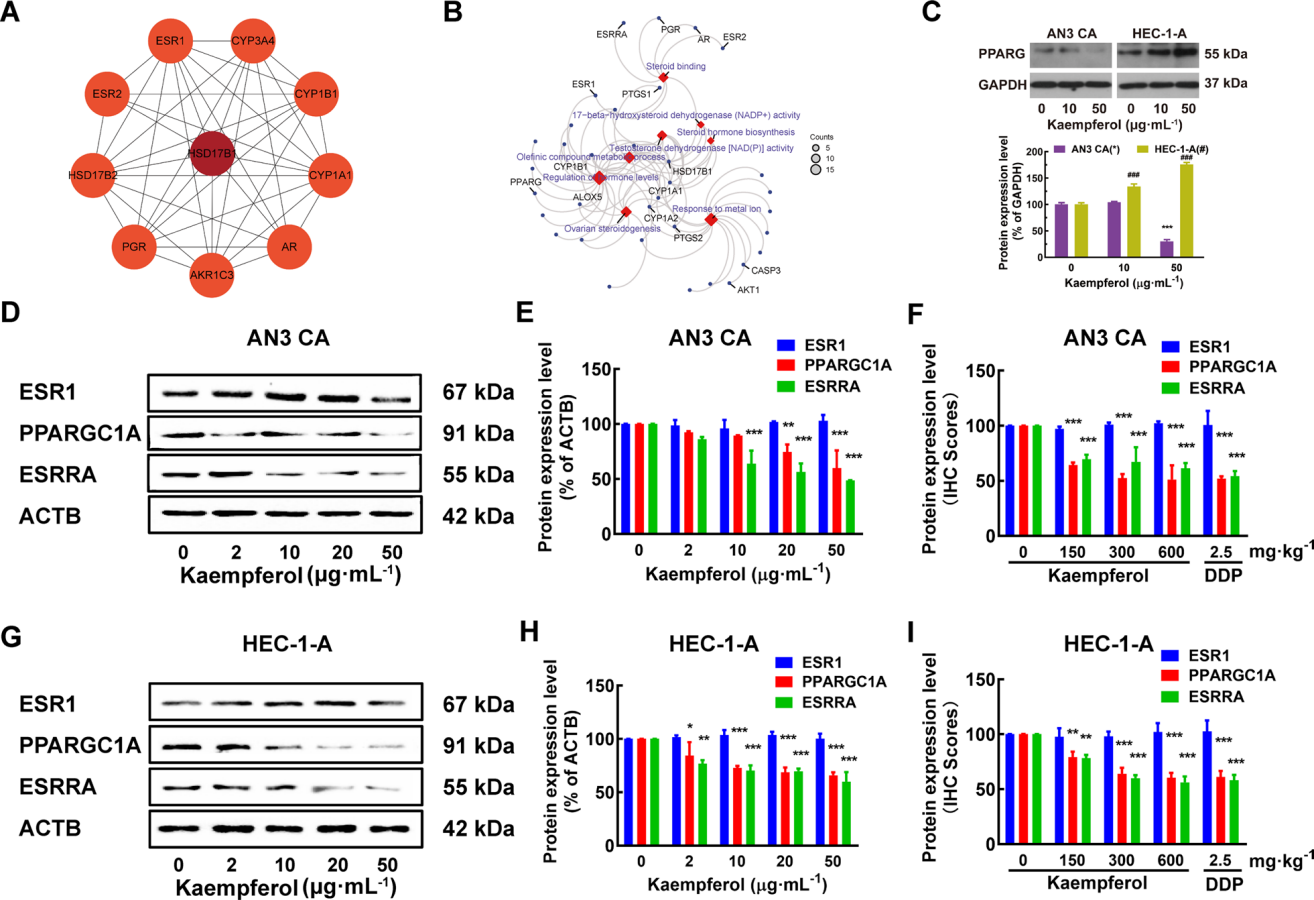
Kaempferol modulated estrogen metabolism pathways and differentially regulates PPARG expression in EC cells of different ER subtypes. **A**–**B** HSD17B1 and HSD17B1-associated genes, such as ESRRA, PPARG, and ESR1, are involved in several estrogen metabolism pathways, such as steroid binding, 17- beta-hydroxysteroid dehydrogenase (NADP+) activity, steroid hormone biosynthesis, and regulation of hormone levels. **C** Kaempferol suppressed the expression of PPARG in ER-positive AN3 CA and promoted the expression of PPARG in ER-negative HEC-1-A. **D**–**I** Kaempferol suppressed the expression of PPARGC1A and ESRRA in both AN3 CA (**D**–**F**) and HEC-1-A cells (**G**–**I**), without modulating ESR1. Western blotting (**D**–**E** and **G**–**H**) and the IHC scores (**F** and **I**) confirmed the differential expression of PPARGC1A and ESRRA. Results are presented as means and SDs. Compared with the negative control, ^*, #^*P* < 0.05, ^**, ##^*P* < 0.01, ^***, ###^*P* < 0.001

HSD17B1 and HSD17B1-associated genes, such as ESR1, ESRRA, PPARG, AKT1, and AKR1C1\2\3, were involved in several estrogen metabolism pathways, such as steroid binding, 17-beta-hydroxysteroid dehydrogenase (NADP+) activity, steroid hormone biosynthesis, and regulation of hormone levels (Figs. 5A–B). The molecular basis of the effects of kaempferol treatment was evaluated. RT-qPCR analyses were used to determine the effect of kaempferol on ESRRA, PPARG, AKT1, and AKR1C1\2\3 mRNA expression (Additional file 4: Figs. S4C–D). Immunoblotting and densitometric analyses were used to determine the effect of kaempferol on PPARG, ESR1, PPARGC1A, and ESRRA expression. Kaempferol promoted the mRNA expression of AKT1 in both ER subtypes EC cells and suppressed the mRNA expression of AKR1C1\2\3 in ER-positive AN3 CA cells (Additional file 4: Fig. S4C-D, *P* < 0.05). Kaempferol suppressed the expression of PPARG in ER-positive AN3 CA and promoted the expression of PPARG in ER-negative HEC-1-A and KLE (Fig. 5C and Additional file 4: Fig. S4D, *P* < 0.05). Kaempferol decreased the activation of PPARGC1A and ESRRA in AN3 CA and HEC-1-A cells but did not affect ESR1 (Figs. 5D–E, G–H, and Additional file 4: Fig. S4C, *P* < 0.05). Immunohistochemical staining was used to measure the expression levels of ESR1, PPARGC1A, and ESRRA in long-term treatments with kaempferol. Tissues from the treated group exhibited significantly lower levels of PPARGC1A and ESRRA than those from the negative control group (Fig. 5F and I, *P* < 0.05).

## Discussion

The phytoestrogen kaempferol is abundantly available and easily accessible in fruits, vegetables, and tea, and it has been found to have a variety of biological actions, including anti-oxidative and anti-carcinogenic activities, which has promising translational medical applications [47]. In the present study, we used an integrated approach of network pharmacology, molecular docking, and experimental verification to uncover kaempferol as an effective modulator of HSD17B1 for the treatment of EC both in vitro and in vivo. Interestingly, a further novel finding is that the sensitivity of kaempferol was positively correlated with HSD17B1 expression in EC cells.

The pathological subtypes of EC have been characterized by unique histological manifestations and distinct biological behaviors. While the TCGA-inspired EC molecular classification has shown initial promise, it is worth noting that none of the EC cell lines fully represent any one of the four TGCA‑based categories [48, 49]. Moreover, it is probable that a single cell line or tumor would exhibit features of two or more categories, rendering TGCA‑based categorizations challenging [50]. Furthermore, most studies continue to use the classical Bokhman’s or WHO histologic classification system, type I (estrogen-related EC) versus type II (non-estrogen-related EC), or no histological classification system at all [49]. Previous research has also shown a significant correlation between the subtypes of different classification systems and hormone-related treatment responses [19, 51, 52]. Hence, we used well-characterized cell lines from type I (AN3 CA and Ishikawa) and type II (HEC-1-A, HEC-1-B, and KLE) tumors as EC models to examine the molecular genetics and processes underlying their response to kaempferol [46, 53, 54].

Previous in vitro studies have examined the anti-endometrial cancer effects of kaempferol without exploring potential differences in its effects on different ER subtypes [10, 11]. In contrast, our study found that kaempferol could inhibit not only the growth of tumors but also the potential for metastatic tumor formation, regardless of ER subtype, although the extent of inhibition may vary depending on the specific ER subtype of the EC cell line (Fig. 1). These partly explain a positive correlation between *ESRRA* mRNA expression and the International Federation of Gynecology and Obstetrics stage as well as myometrial invasion [55]. Furthermore, our study provides initial in vivo validation of the effects of kaempferol on different ER subtypes (Fig. 2). The acute and chronic toxicity of kaempferol as a potential therapeutic agent was evaluated in both healthy cell lines and human EC xenograft models (Figs. 1B, 2H and Additional file 1: Figs. S1B-E). In contrast, significant changes in organ coefficients were observed in DDP-treated animals (Fig. 2H). These findings suggest that kaempferol is a safe potential therapeutic agent for the treatment of EC [56].

Moreover, the inhibited tumor growth observed in the kaempferol-treated group was attributed to increased apoptosis, as confirmed by HE staining and TUNEL assay (Fig. 2). We demonstrated that kaempferol-induced apoptosis and decreased necrosis in AN3 CA and HEC-1-A tumors. Kaempferol increased the levels of cleaved caspase-9 (CASP9) and caspase-3 (CASP3). The mechanism of kaempferol-induced apoptosis was associated with the activation of cell surface death receptors and the mitochondrial pathway [57]. Additionally, kaempferol treatment resulted in a dose-dependent alteration in the cell cycle of EC cells, with an increase in the S phase, a decrease in the G_0_/G_1_ phase, and a prolongation of the G_2_/M phase (Additional file 2: Fig. S2). This effect could be attributed to the inhibition of ESRRA and its downstream regulation of p21 and hypophosphorylated Rb, which control the G_1_/S checkpoint and cellular energetics during the cell cycle [58, 59]. Taken together, these results suggest that kaempferol exerts its anti-cancer effects in EC cells through the induction of apoptosis and modulation of the cell cycle, which may be mediated by the inhibition of ESRRA.

EC patients with a high HSD17B1 expression level had significantly worse OS in the TCGA database (Fig. 4E), which suggests HSD17B1 may play an important role in the development and progression of EC and could serve as a potential prognostic biomarker for EC [60, 61]. HSD17B1 and CYP1A1 are both involved in estrogen metabolism and have been implicated in the development and progression of EC. HSD17B1 catalyzes the conversion of estrone to estradiol, which is the most potent form of estrogen, and also plays a role in the conversion of androstenedione to testosterone. CYP1A1, on the other hand, is involved in the metabolism of estrogen and other xenobiotics [62]. The observed low expression of HSD17B1, CYP1A1, and CYP1B1 in ER-negative EC cells, especially in KLE where no expression was detected, indicates that the expression of HSD17B1 is closely associated with ER subtypes. This finding may provide a new insight into the molecular mechanisms underlying ER-negative EC, and suggests that HSD17B1 may contribute to the development and progression of ER-negative EC. The positive correlation between HSD17B1 expression and kaempferol sensitivity, as well as the up-regulation of HSD17B1 with kaempferol treatment, suggests that HSD17B1 may serve as a potential biomarker for predicting the therapeutic response to kaempferol in EC [60].

**Fig. 6:**
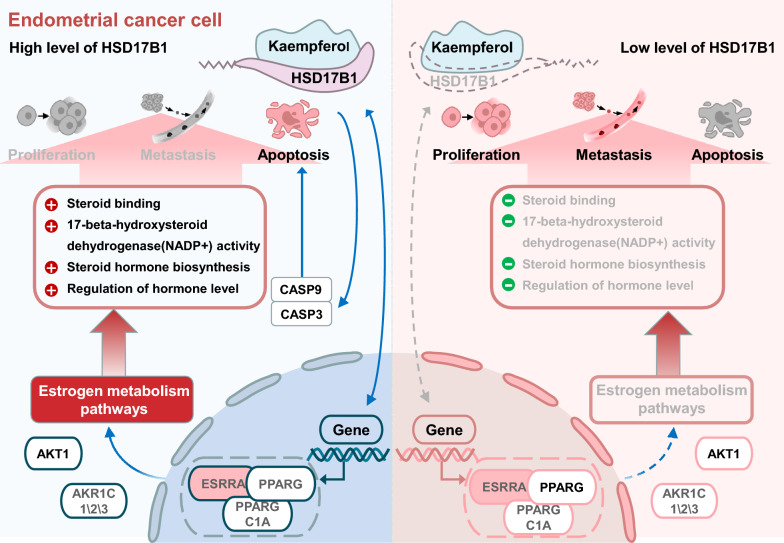
Schematic diagram of the mechanism by which kaempferol induces apoptosis and inhibits growth and metastasis via HSD17B1 in EC cells

EC is a hormone-dependent disease in which estrogen is involved in carcinogenesis and promotes disease progression. However, the inefficacy of hormonal therapy in EC suggests that the disease involves complex estrogen signaling pathways [63, 64]. The crosstalk between ESR1 and ESRRA remains controversial. Previous studies have reported that downregulated ESRRA expression is not associated with ERS1 status, which affects cell proliferation and apoptosis [19]. In this study, we found that kaempferol decreased the expression of ESRRA, but did not affect ESR1 in EC cells. This suggests the existence of complex crosstalk between ESR1 and ESRRA in EC. HSD17B1, which is involved in several estrogen metabolism pathways and associated with ESR1, ESRRA, PPARG, AKT1, and AKR1C1\2\3, was identified as a differentially expressed gene in response to kaempferol treatment. Interestingly, kaempferol upregulated HSD17B1 expression and sensitivity in ER-negative EC cells. The interaction between HSD17B1 and ESRRA, and their involvement in estrogen metabolism is still unknown but may involve the regulation of PPARG and adipocyte differentiation [65]. It was found that kaempferol had differential effects on the expression of PPARG in ER-positive and ER-negative EC cells. Specifically, it suppressed PPARG expression in ER-positive AN3 CA cells, while promoting PPARG expression in ER-negative HEC-1-A and KLE cells (Fig. 5C and Additional file 4: Fig. S4D). Additionally, kaempferol was found to decrease the activation of PPARGC1A and ESRRA in AN3 CA and HEC-1-A cells, without affecting ESR1 (Figs. 5D–E, G–H, and Additional file 4: Fig. S4C). It is important to note that ESRRA and its coactivators, PPARGC1A and PPARG, are central signaling molecules in metabolic pathways that play a role in various aspects of tumor progression, such as rapid growth, proliferation, responses to environmental stress, migration, metastasis, and drug resistance [66]. This may provide additional insight into the potential mechanisms of kaempferol's effects on EC cells (Fig. 6).

This study provides substantial evidence for the anti-tumor activities of kaempferol in both ER subtypes of EC cells and a positive association between HSD17B1 expression and kaempferol’s sensitivity; however, there are several limitations to consider when interpreting the findings presented. The first and most significant limitation is that tumor cell analyses were limited to standard EC cell lines. We believe that the results shown in these cell lines are representative of two ER subtypes based on clinical, histological, and molecular characteristics. However, future studies may expand upon these findings through the use of patient-derived organoids or xenograft models of four TGCA‑based clusters. The second limitation of this study was the lack of a direct demonstration of the mechanism by which HSD17B1 and ESRRA interact. Thus, additional research is needed to more fully elucidate the direct mechanisms of kaempferol's anti-cancer effects and to optimize its pharmacological properties. The pharmacokinetic and toxicological properties of kaempferol also need to be carefully evaluated to ensure its safety and effectiveness in patients.

In conclusion, we found that kaempferol is a promising novel therapeutic candidate for EC. Further studies are warranted to explore the HSD17B1 and ESRRA interaction in the treatment of kaempferol. Our results can aid in the development of new hormonal and molecular-targeted therapeutic agents for treating EC. Further, these results will help to improve the efficiency of the medical translation of phytoestrogens.

## Supplementary Information

**Additional file 1: Fig. S1.** Cytotoxicity of DDP and kaempferol in EC and healthy cells. **A** The percentage of viable cells treated with DDP was significantly lower in ER-positive cells than in ER-negative cells. The viability values for AN3 CA and Ishikawa were 44.99% and 39.49%, respectively, compared to 70.73% and 66.02% for HEC-1-A and HEC-1-B, respectively. Human healthy cells also showed significantly higher cell viability than ER-positive cells when treated with DDP at a concentration of 2 μg·mL^−1^ for 48 h. The viability values were 71.02%, 66.63%, 51.70%, and 75.53% for MRC-5, CCD 841 CoN, WRL 68, and monocytes, respectively. **B**–**E** Kaempferol showed low cytotoxicity towards peripheral-venous-blood extracted human monocytes **B**, MRC-5 **C**), WRL 68 **D**), and CCD 841 CoN **E** cell lines incubated with increasing doses of kaempferol (0, 2, 5, 10, 20, and 50 μg·mL^−1^) for 48 h. Compared with the cell viability of HEC-1-A cells, ^*^P < 0.05; ^**^P < 0.01; ^***^P < 0.001.

**Additional file 2: Fig. S2.** Kaempferol-induced effected in apoptosis and cell cycle in EC cells. **A** Apoptosis of EC cells was analyzed by flow cytometry using Annexin V and PI markers for apoptosis. **B** Kaempferol decreased the fraction of AN3 CA’s cell cycle in G_0_/G_1_ phase in a dose-dependent manner and increased the fraction in the S phase and G_2_/M phase at a concentration of 10 μg·mL^−1^ and 20 μg·mL^−1^, respectively.

**Additional file 3: Fig. S3.** Kaempferol reduced tumor volume without causing adverse effects in both ER subtypes of mouse xenograft models. **A**–**B** Treatment with kaempferol by intragastric administration as well as DDP significantly reduced tumor volume in both ER subtypes of mouse xenograft models when compared to the vehicle. **C**–**D** No adverse effects or significant changes in body weight was observed in AN3 CA **C** or HEC-1-A **D** xenograft models. Compared with the negative control, ^*^P < 0.05, ^**^P < 0.01,^***^P < 0.001. 

**Additional file 4: Fig. S4.** The mRNA expression level of CYP1A1 **A**, CYP1B1 **B**, ESRRA **C**, PPARG **D**, AKT1 **E**, and AKR1C1\2\3 **F** in EC cells with kaempferol treatment. Compared with the negative control, ^*^P < 0.05, ^**^P < 0.01, ^***^P < 0.001.

## Data Availability

The datasets used and/or analysed during the current study are available from the corresponding author on reasonable request.
