# Correction to: An integrated approach of network pharmacology, molecular docking, and experimental verification uncovers kaempferol as the effective modulator of HSD17B1 for treatment of endometrial cancer

**DOI:** 10.1186/s12967-026-07698-x

**Published:** 2026-02-13

**Authors:** Guan‑Yu Ruan, Li‑Xiang Ye, Jian‑Song Lin, Hong‑Yu Lin, Li‑Rui Yu, Cheng‑Yan Wang, Xiao‑Dan Mao, Shui-Hua Zhang, Peng‑Ming Sun

**Affiliations:** 1 Laboratory of Gynecologic Oncology, Fujian Maternity and Child Health Hospital, College of Clinical Medicine for Obstetrics & Gynecology and Pediatrics, Fujian Medical University, Fuzhou, 350001 Fujian People’s Republic of China; 2 Fujian Key Laboratory of Women and Children’s Critical Diseases Research, Fujian Maternity and Child Health Hospital, Fuzhou, 350001 Fujian People’s Republic of China; 3 Fujian Clinical Research Center for Gynecologic Oncology, Fujian Maternity and Child Health Hospital (Fujian Obstetrics and Gynecology Hospital), Fuzhou, 350001 Fujian People’s Republic of China; 4 Fujian Center for Safety Evaluation of New Drugs, Fujian Medical University, No.1 Xue Fu Bei Road, University Town, Fuzhou, 350001 Fujian People’s Republic of China; 5 Department of Pathology, Fujian Maternity and Child Health Hospital, College of Clinical Medicine for Obstetrics & Gynecology and Pediatrics, Fujian Medical University, Fuzhou, 350001 Fujian People’s Republic of China; 6 Collage of Pharmacy, Fujian Medical University, Fuzhou, 351004 Fujian People’s Republic of China; 7 Animal Research Institute, Fujian Medical University, Fuzhou, 351004 Fujian People’s Republic of China

**Correction to: Journal of Translational Medicine (2023)**

10.1186/s12967-023-04048-z

The authors wish to correct the following errors in the original publication [1]. These errors were the result of inadvertent mistakes during figure assembly and manuscript preparation and do not affect the results or conclusions of the study.

1. Correction to Fig. 1F:

In the original version of Fig. 1F, the GAPDH loading control panels for the AN3 CA and HEC-1-A cell lines were inadvertently swapped. To resolve any potential confusion and to present the data with the highest clarity, the panels for Cleaved CASP 3, Cleaved CASP 9, and GAPDH in Fig. 1F have been replaced with data from an independent biological replicate. The corrected figure is presented below.

**Figure:**
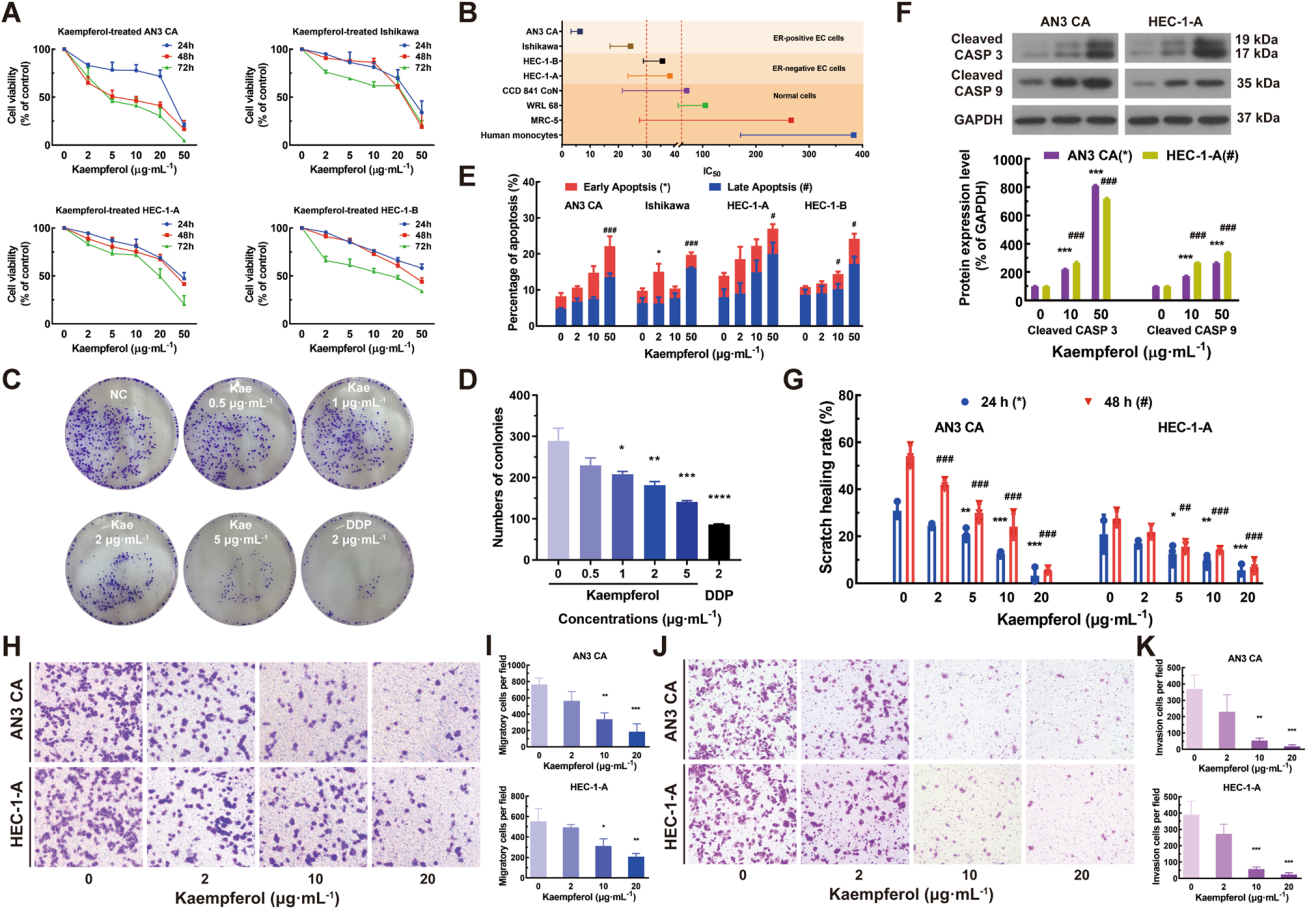

2. Correction to Fig. 2F:

In the original version of Fig. 2F, the immunohistochemistry (IHC) image for the CASP9 group (kaempferol 0 mg·kg⁻¹) treatment in HEC-1-A group was inadvertently misused from the CASP3 group (kaempferol 150 mg·kg⁻¹ treatment). The figure has been replaced with the correct image for the CASP9 group from our original dataset. The corrected figure is presented below.

**Figure:**
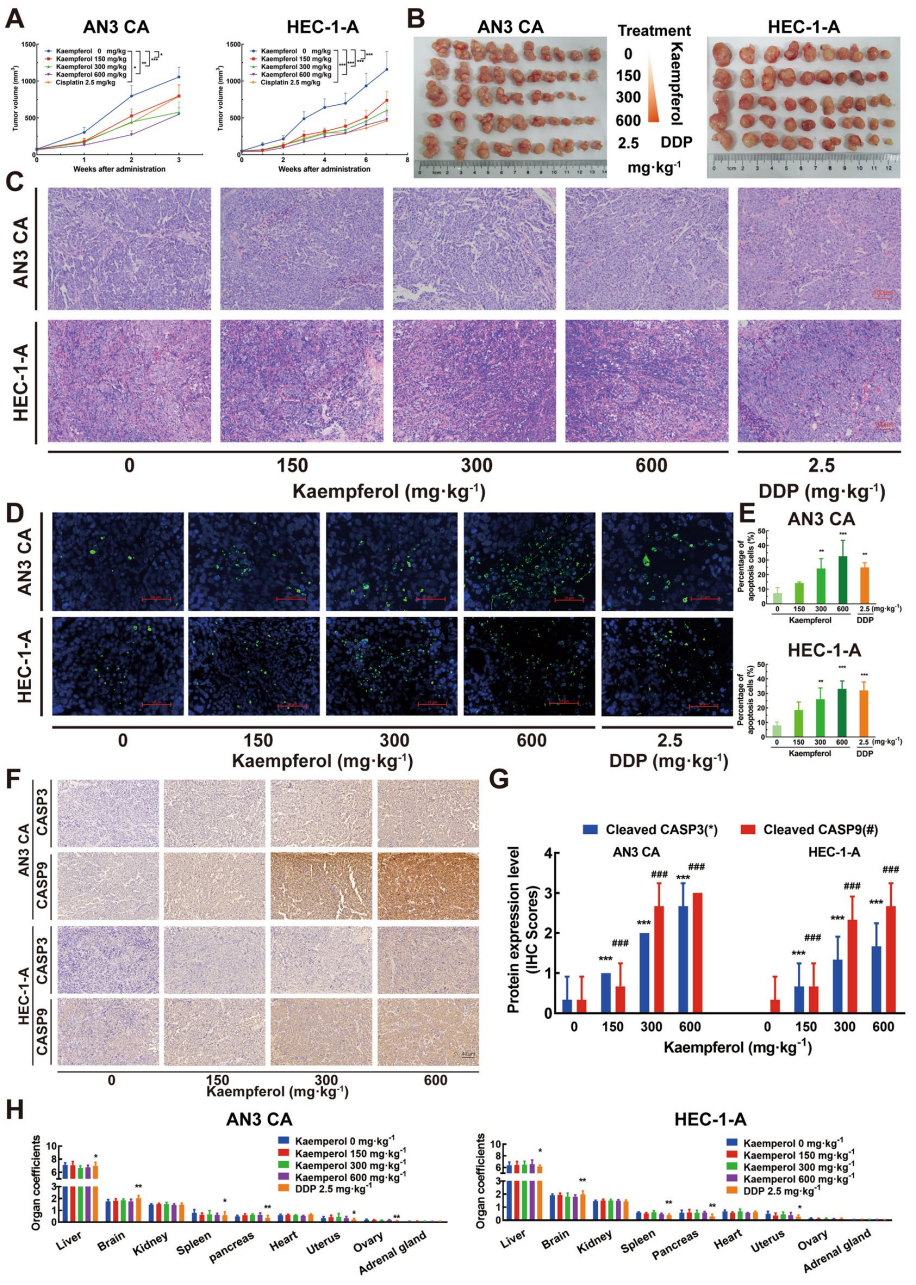

3. Correction to Fig. 4B:

To address the concern regarding the presentation of control data and to ensure each figure is supported by a unique internal control, the panels for HSD17B1 and GAPDH in Fig. 4B have been replaced with data from an independent biological replicate. This change eliminates any perceived redundancy and strengthens the visual presentation of the results. The corrected figure is presented below.

**Figure:**
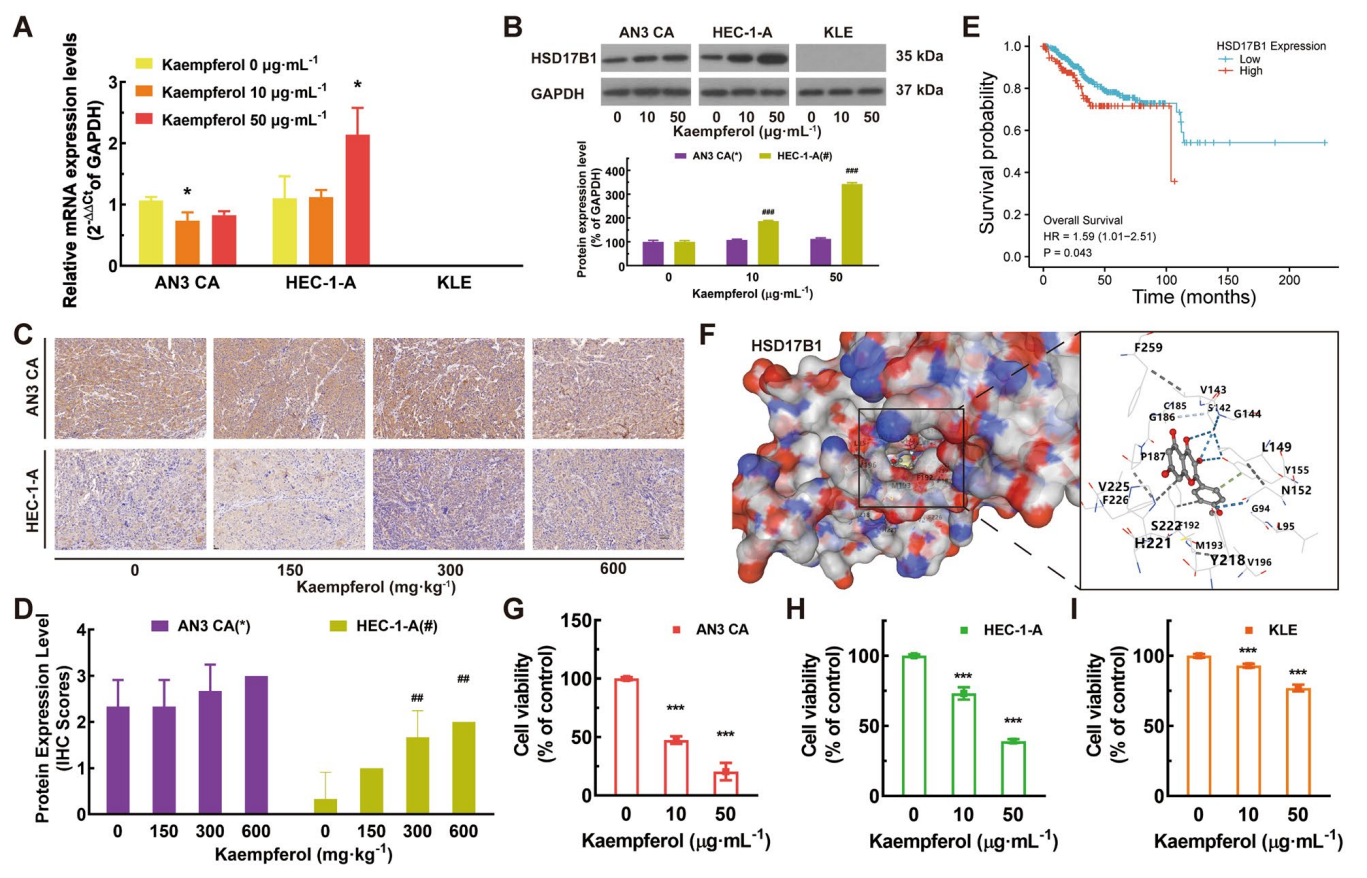

4. Correction to Affiliations:

The first and fifth institutional affiliations in the author details were listed incorrectly. The correct affiliations are as follows:

1 Laboratory of Gynecologic Oncology, Fujian Maternity and Child Health Hospital, College of Clinical Medicine for Obstetrics & Gynecology and Pediatrics, Fujian Medical University, Fuzhou 350,001, Fujian, People’s Republic of China.

5 Department of Pathology, Fujian Maternity and Child Health Hospital, College of Clinical Medicine for Obstetrics & Gynecology and Pediatrics, Fujian Medical University, Fuzhou 350,001, Fujian, People’s Republic of China.

The original article [1] has been updated to reflect these changes.

The authors sincerely apologize for these errors and any confusion they may have caused. The scientific conclusions of the study remain fully supported by the data.

