# Supplementary figures and images for: An integrated approach of network pharmacology, molecular docking, and experimental verification uncovers kaempferol as the effective modulator of HSD17B1 for treatment of endometrial cancer
Source: J Transl Med. 2023 Mar 17;21:204. doi: 10.1186/s12967-023-04048-z (PMC10022092; doi:10.1186/s12967-023-04048-z)

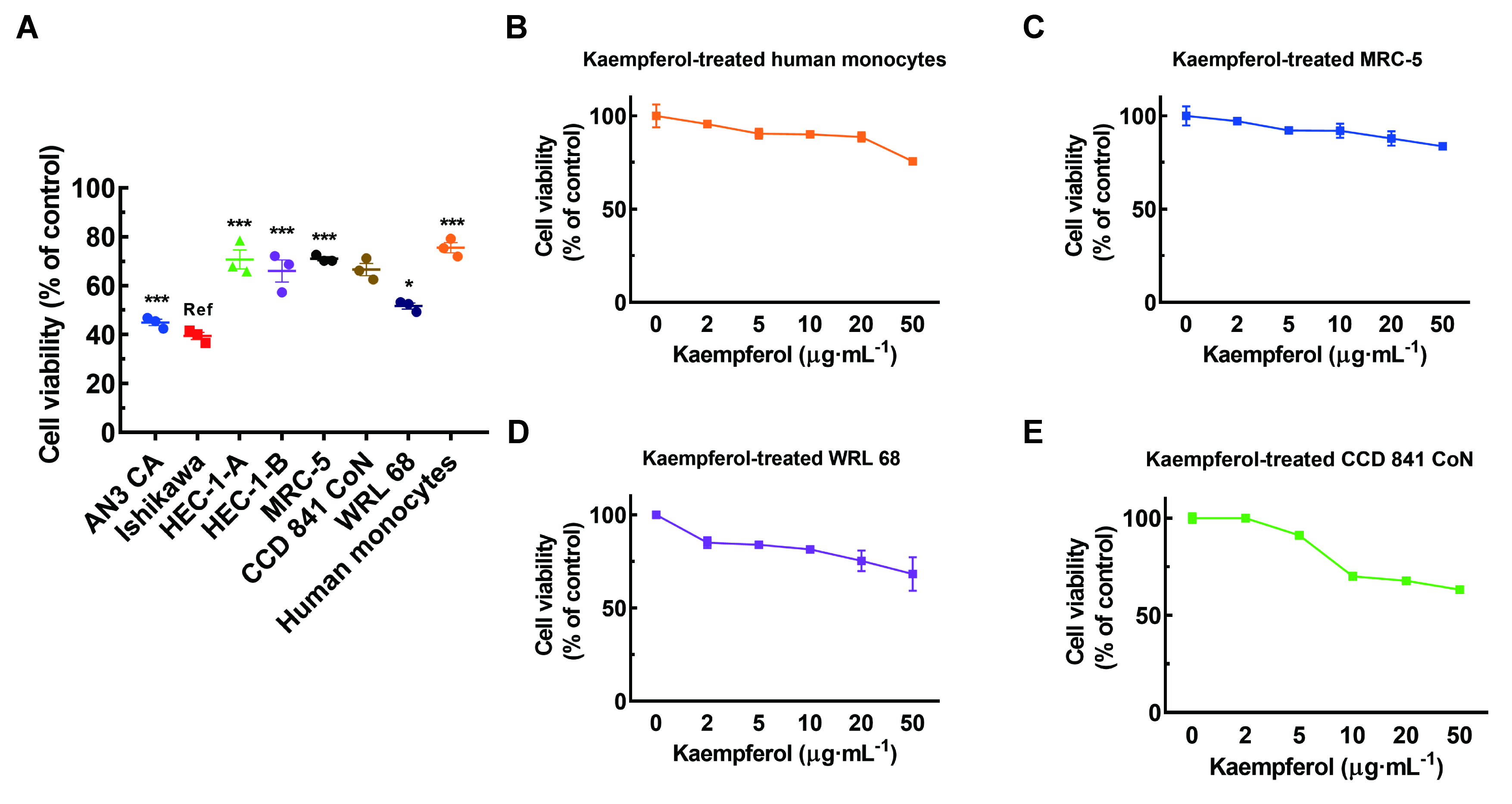

Supplement: Supplementary file 1 — Additional file 1: Fig. S1. Cytotoxicity of DDP and kaempferol in EC and healthy cells. A The percentage of viable cells treated with DDP was significantly lower in ER-positive cells than in ER-negative cells. The viability values for AN3 CA and Ishikawa were 44.99% and 39.49%, respectively, compared to 70.73% and 66.02% for HEC-1-A and HEC-1-B, respectively. Human healthy cells also showed significantly higher cell viability than ER-positive cells when treated with DDP at a concentration of 2 μg·mL−1 for 48 h. The viability values were 71.02%, 66.63%, 51.70%, and 75.53% for MRC-5, CCD 841 CoN, WRL 68, and monocytes, respectively. B–E Kaempferol showed low cytotoxicity towards peripheral-venous-blood extracted human monocytes B, MRC-5 C), WRL 68 D), and CCD 841 CoN E cell lines incubated with increasing doses of kaempferol (0, 2, 5, 10, 20, and 50 μg·mL−1) for 48 h. Compared with the cell viability of HEC-1-A cells, *P < 0.05; **P < 0.01; ***P < 0.001. [file 12967_2023_4048_MOESM1_ESM.tif]

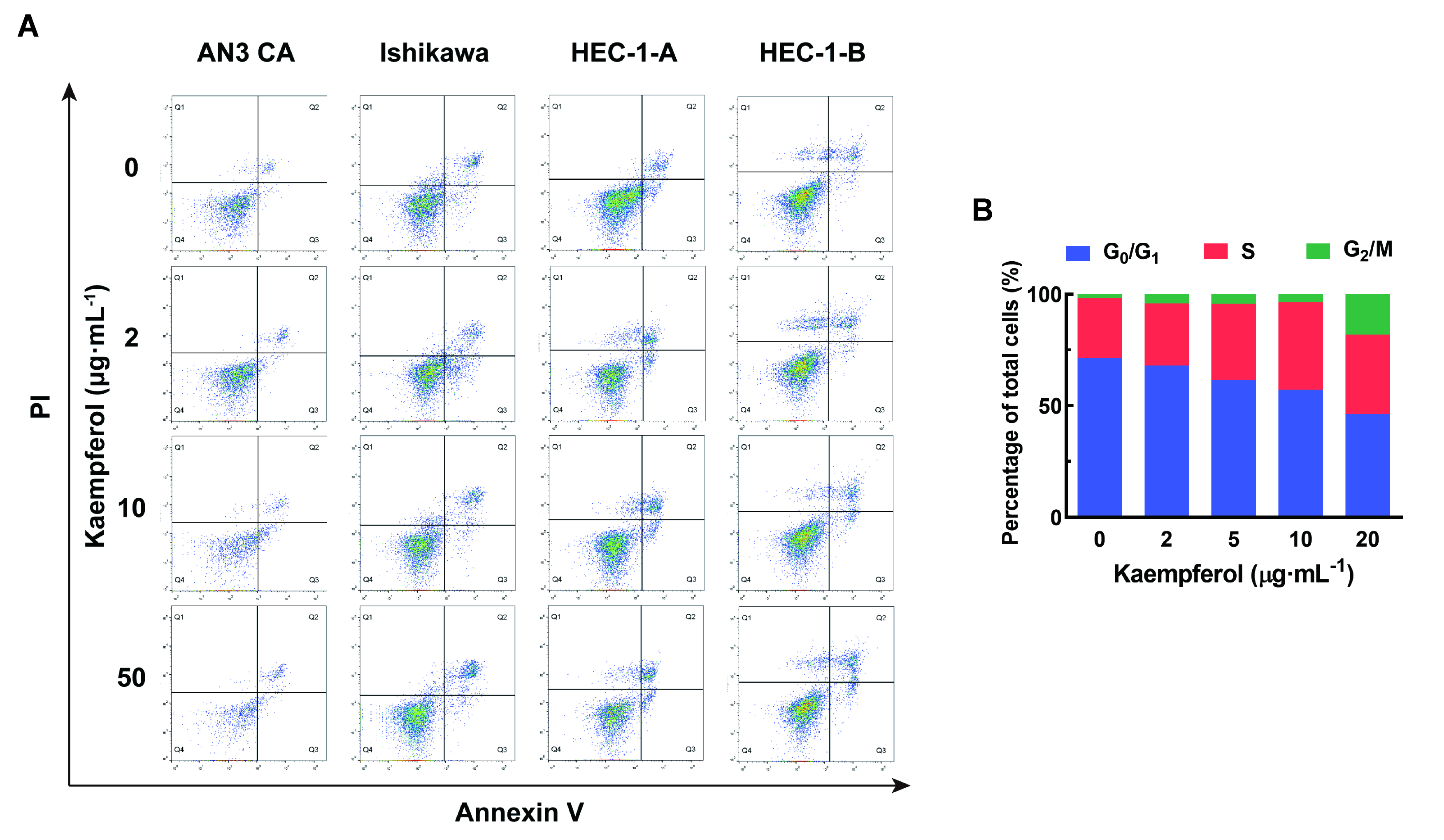

Supplement: Supplementary file 2 — Additional file 2: Fig. S2. Kaempferol-induced effected in apoptosis and cell cycle in EC cells. A Apoptosis of EC cells was analyzed by flow cytometry using Annexin V and PI markers for apoptosis. B Kaempferol decreased the fraction of AN3 CA’s cell cycle in G0/G1 phase in a dose-dependent manner and increased the fraction in the S phase and G2/M phase at a concentration of 10 μg·mL−1 and 20 μg·mL−1, respectively. [file 12967_2023_4048_MOESM2_ESM.tif]

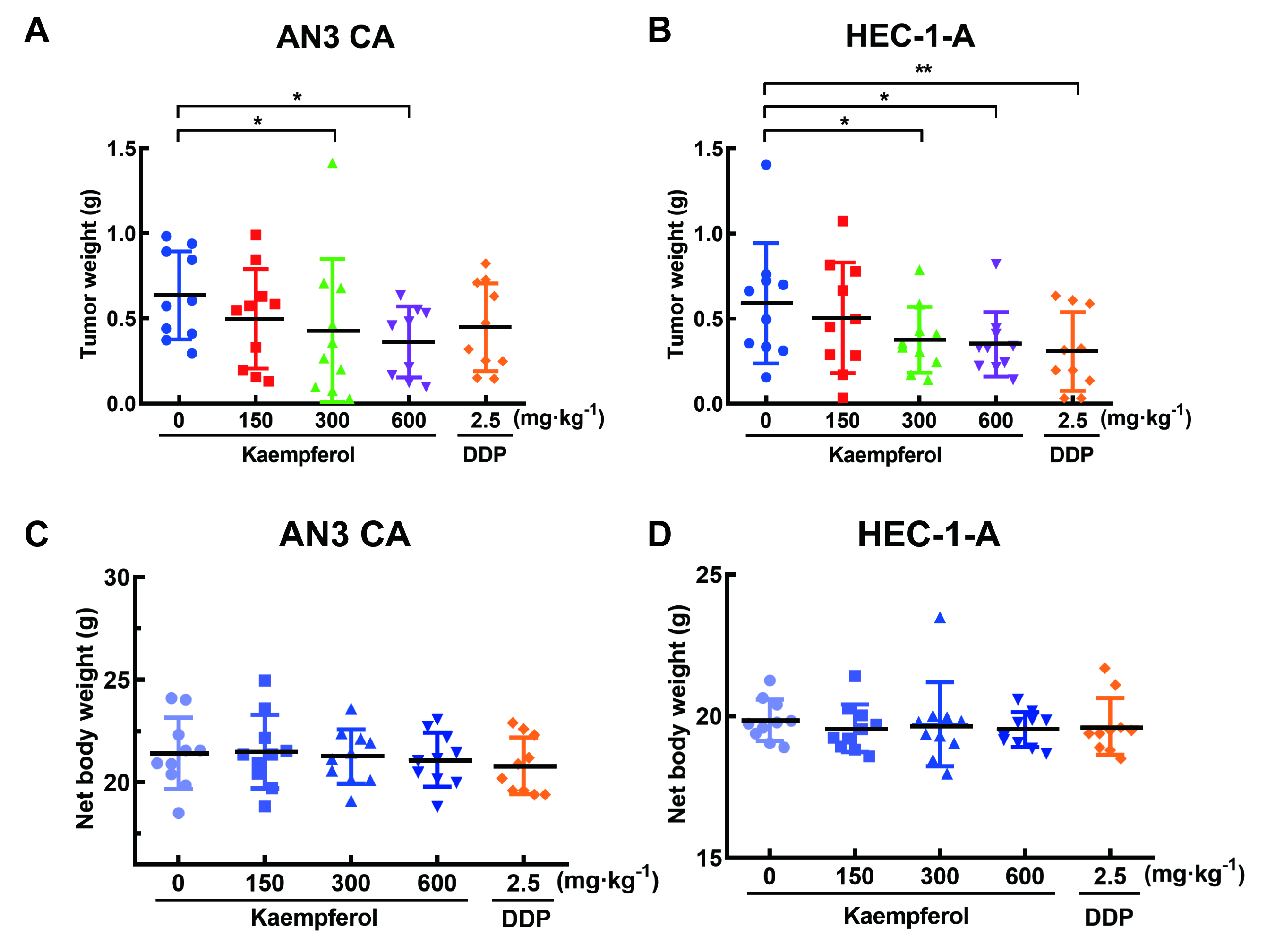

Supplement: Supplementary file 3 — Additional file 3: Fig. S3. Kaempferol reduced tumor volume without causing adverse effects in both ER subtypes of mouse xenograft models. A–B Treatment with kaempferol by intragastric administration as well as DDP significantly reduced tumor volume in both ER subtypes of mouse xenograft models when compared to the vehicle. C–D No adverse effects or significant changes in body weight was observed in AN3 CA C or HEC-1-A D xenograft models. Compared with the negative control, *P < 0.05, **P < 0.01,***P < 0.001. [file 12967_2023_4048_MOESM3_ESM.tif]

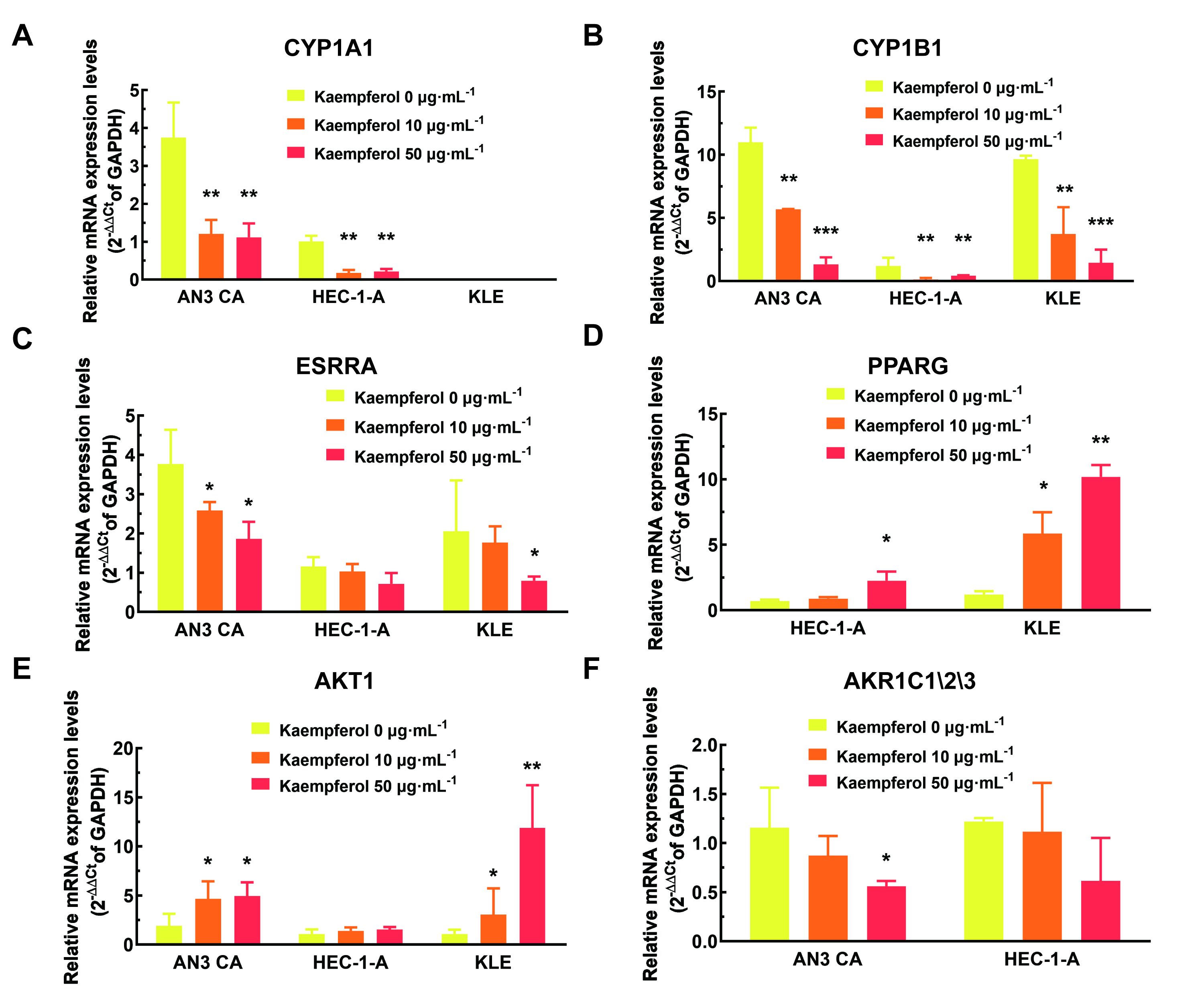

Supplement: Supplementary file 4 — Additional file 4: Fig. S4. The mRNA expression level of CYP1A1 A, CYP1B1 B, ESRRA C, PPARG D, AKT1 E, and AKR1C1\2\3 F in EC cells with kaempferol treatment. Compared with the negative control, *P < 0.05, **P < 0.01, ***P < 0.001. [file 12967_2023_4048_MOESM4_ESM.tif]
